# Mutation testing with hyperproperties

**DOI:** 10.1007/s10270-020-00850-1

**Published:** 2021-04-01

**Authors:** Andreas Fellner, Mitra Tabaei Befrouei, Georg Weissenbacher

**Affiliations:** 1 AIT Austrian Institute of Technology, Seibersdorf, Austria; 2 TU Wien, Vienna, Austria

## Abstract

We present a new method for model-based mutation-driven test case generation. Mutants are generated by making small syntactical modifications to the model or source code of the system under test. A test case kills a mutant if the behavior of the mutant deviates from the original system when running the test. In this work, we use hyperproperties—which allow to express relations between multiple executions—to formalize different notions of *killing* for both deterministic as well as non-deterministic models. The resulting hyperproperties are universal in the sense that they apply to arbitrary reactive models and mutants. Moreover, an off-the-shelf model checking tool for hyperproperties can be used to generate test cases. Furthermore, we propose solutions to overcome the limitations of current model checking tools via a model transformation and a bounded SMT encoding. We evaluate our approach on a number of models expressed in two different modeling languages by generating tests using a state-of-the-art mutation testing tool.

## Introduction

The ever rising complexity of systems demands automated methods for creating high quality test suites. In this work, we present such a method by solving mutation-based test generation via hyperproperty model checking. Furthermore, we lay the theoretical foundation for future research in the area by showing that strong mutation killing is a hyperproperty and by carefully examining the role of non-determinism for mutation analysis.

A mutant is a small syntactic modification of some description of the system under test. The aim of mutation-based test generation is to construct tests that reveal these modifications, where revealing either means showing a difference in internal state (weak mutation [39]), or showing a difference in observable output (strong mutation [16]). The approach is based on two assumptions: (a) the *competent programmer hypothesis* [16], which states that implementations are typically close-to-correct, and (b) the *coupling effect* [49], which states that a test suite’s ability to detect simple errors (and mutations) is indicative of its ability to detect complex errors. High mutation coverage was shown to correlate well with high quality test suites [6].

In model-based testing, test cases are generated from an ideal abstraction of the system under test, for example a specification. Tests created in this way can verify or reject whether a system under test is in fact an implementation of that ideal abstraction. Model checking has been applied successfully in this context [35]. The idea is that tests are counter-examples to encodings of coverage criteria. Even though this success was partially carried over to mutation-based test generation [36, 50], strong mutation analysis does not quite fit into the framework of classical model checking. In our work, we explore the reason for this phenomenon, namely that strong mutation killability is a property reasoning over multiple traces at once, i.e. it is a hyperproperty, in contrast to classical trace-properties that reason over single traces.

Hyperproperties are an emerging field in automated reasoning that studies properties over multiple traces [19]. Its classical field of application is security analysis, where properties such as non-interference are expressed as hyperproperties. We present a novel application for that field and a powerful test case generation method that can be readily applied, as we demonstrate in our experimental evaluation on two different modeling formalisms using off-the-shelf tools.

In summary, the main contributions of our paper are as follows:

- An encoding of mutation killability in HyperLTL, a logic for hyperproperties.
- A careful study of the role of non-determinism in mutation analysis and two novel distinctions of mutation killability: potential and definite.
- Practical solutions for test generation from non-deterministic models.
- An experimental evaluation of mutation-based test case generation via HyperLTL model checking, using multiple modeling formalisms and leveraging an off-the-shelf toolchain.

This paper is based on [26]. On top of textual improvements and an extended elaboration of related work, this version includes the following new contributions:

- A HyperCTL* encoding of tests with inconclusive output information.
- HyperLTL encodings of killability for mixed determinism cases.
- An encoding of test generation for non-determinisitic models as a bounded SMT satisfiability problem with a proof of concept demonstration.
- Detailed proofs of all propositions and lemmas.

The rest of the paper is organized as follows: We conclude the introduction by providing a running example. In Sect. 2, we present our system model and provide the necessary concepts of HyperLTL. In Sect. 3, we discuss mutation analysis in our setting and define potential and definite killing of mutants. In Sect. 4, we provide the HyperLTL encodings of both types of killing and multiple settings in terms of presence or absence of non-determinism, as well as a HyperCTL* encoding of tests with inconclusive output information. In Sect. 5, we discuss handling of non-deterministic models in practice via a transformation to controllable non-determinism or a bounded SMT encoding. In Sect. 6, we present an experimental evaluation of our methods. Finally, in Sect. 7, we discuss related work and conclude in Sect. 8.

**Fig. 1:**
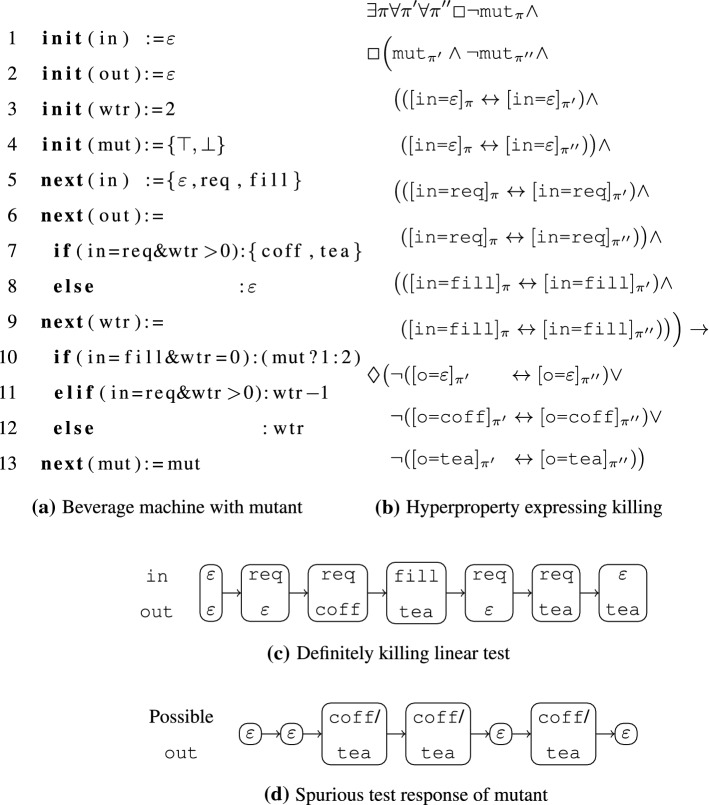
Beverage machine running example

*Running example.* We illustrate the main concepts of our work in Fig. 1. We present the main intuitions here, while the concepts used in the example will be introduced in detail throughout this work.

Figure 1a shows the SMV [46] model of a beverage machine, which non-deterministically serves coff (coffee) or tea after input req (request), assuming that there is still enough wtr (water) in the tank. Water can be refilled with input fill. The symbol $\varepsilon$ represents absence of input and output, respectively.

The code in Fig. 1a includes the variable mut (initialized non-deterministically in line 4), which enables the activation of a mutation in line 10. The mutant refills 1 unit of water only, whereas the original model fills 2 units.

Figure 1b states a hyperproperty over the inputs and outputs of the model formalizing that the mutant can be killed *definitely* (i.e., independently of non-deterministic choices). Figure 1c shows a linear test, i.e. a sequence of inputs and outputs, that is a witness for this claim. The test requests two drinks after filling the tank. For the mutant, the output following the second request after filling the tank must be $\varepsilon$, which is different from the prescribed output $\texttt {tea}$, as indicated in Fig. 1d, which shows all possible output sequences of the mutant for the given test.

## Preliminaries

This section introduces symbolic transition systems as our formalisms for representing discrete reactive systems and provides the syntax and semantics of HyperLTL, a logic for hyperproperties.

### System model

A symbolic transition system (STS) is a tuple ${\mathcal {S}} = \langle {\mathcal {I}},{\mathcal {O}},{\mathcal {X}},\alpha ,\delta \rangle$, where ${\mathcal {I}},{\mathcal {O}},{\mathcal {X}}$ are finite sets of input, output, and state variables, $\alpha$ is a formula over ${\mathcal {X}} \cup {\mathcal {O}}$ (the initial conditions predicate), and $\delta$ is a formula over ${\mathcal {I}} \cup {\mathcal {O}} \cup {\mathcal {X}} \cup {\mathcal {X}} '$ (the transition relation predicate), where ${\mathcal {X}} ' = \{x' \mid x \in {\mathcal {X}} \}$ is a set of primed variables representing the successor states. An input *I*, output *O*, state *X*, and successor state $X'$, respectively, is a mapping of ${\mathcal {I}},{\mathcal {O}}$, ${\mathcal {X}}$, and ${\mathcal {X}} '$, respectively, to values in a range that includes the elements $\top$ and $\bot$ (representing true and false, respectively). We call an STS *finite* if the range of values is finite. A tuple (*I*, *O*, *X*) of input *I*, output *O* and state *X* is called a *system state*. The set of all system states is denoted by ${\mathcal {Y}}$. For some variable mapping *Q*, $Q\vert _{{{\mathcal {V}}}}$ denotes the restriction of the domain of *Q* to the variables ${\mathcal {V}}$. Given a mapping *Q* and variable $v\in {\mathcal {V}}$, *Q*(*v*) denotes the value of *v* in *Q* (if defined) and $Q[v \mapsto x]$ denote *Q* with *v* mapped to value *x*.

We assume that the initial conditions- and transition relation predicate are defined in a logic that includes standard Boolean operators $\lnot$, $\wedge$, $\vee$, $\rightarrow$, and $\leftrightarrow$. We omit further details, as our results do not depend on a specific formalism. We write $X,O \models \alpha$ and $I,O,X,X' \models \delta$ to denote that $\alpha$ and $\delta$ evaluate to true under an evaluation of inputs *I*, outputs *O*, states *X*, and successor states $X'$. We assume that every STS has a distinct output $O_{\varepsilon }$, representing absence of output.

A state *X* with output *O* such that $X,O \models \alpha$ are an *initial state* and *initial output*. A state *X* has a transition with input *I* to its *successor state* $X'$ with output *O* iff $I,O,X,X' \models \delta$, denoted by $X \xrightarrow {I,O} X'$. A *trace* of ${\mathcal {S}}$ is a sequence of system states $\left\langle (I_0,O_0,X_0), (I_1,O_1,X_1), (I_2,O_2,X_2), \ldots \right\rangle \in {\mathcal {Y}}^{\omega }$ such that $X_0,O_0\models \alpha$ and $\forall j \ge 0\,.\, X_j \xrightarrow {I_j,O_{j+1}} X_{j+1}$. We require that every system state has at least one successor, therefore all traces of ${\mathcal {S}}$ are infinite. We denote by ${\mathcal {T}} ({\mathcal {S}})$ the set of all traces of ${\mathcal {S}}$. Given a trace $p = \left\langle (I_0,O_0,X_0), (I_1,O_1,X_1), \ldots \right\rangle$, we write *p*[*j*] for $(I_j,O_j,X_j)$, *p*[*j*, *l*] for $\langle (I_j,O_j,X_j),\ldots ,(I_l,O_l,X_l) \rangle$, $p[j,\infty ]$ for $\langle (I_j,O_j,X_j),\ldots \rangle$ and $p\vert _{{{\mathcal {V}}}}$ to denote $\left\langle ({I_0}\vert _{{{\mathcal {V}}}},{O_0}\vert _{{{\mathcal {V}}}},{X_0}\vert _{{{\mathcal {V}}}}), ({I_1}\vert _{{{\mathcal {V}}}},{O_1}\vert _{{{\mathcal {V}}}},{X_1}\vert _{{{\mathcal {V}}}}), \ldots \right\rangle$. We lift restriction to sets of traces *T* by defining $T\vert _{{{\mathcal {V}}}}$ as $\{p\vert _{{{\mathcal {V}}}} \mid t \in T\}$.

${\mathcal {S}}$ is *deterministic* iff there is a unique pair of an initial state and initial output and for each state *X* and input *I*, there is at most one state $X'$ with output *O*, such that $X\xrightarrow {I,O} X'$. Otherwise, the model is *non-deterministic*.

In the following, we presume the existence of sets of atomic propositions $\mathsf {AP} =\{\mathsf {AP} _{{\mathcal {I}}}\cup \mathsf {AP} _{{\mathcal {O}}}\cup \mathsf {AP} _{{\mathcal {X}}}\}$ (intentionally kept abstract)^1^ and sets $\mathsf {AP} (I) \subseteq \mathsf {AP} _{{\mathcal {I}}}, \mathsf {AP} (O) \subseteq \mathsf {AP} _{{\mathcal {O}}}, \mathsf {AP} (X) \subseteq \mathsf {AP} _{{\mathcal {X}}}$ that uniquely characterize input *I*, output *O*, and state *X*. For a system state (*I*, *O*, *X*), we define $\mathsf {AP} (I,O,X) {\mathop {=}\limits ^{\tiny def }}\mathsf {AP} (I) \cup \mathsf {AP} (O) \cup \mathsf {AP} (X)$. For a trace $p = \left\langle (I_0,O_0,X_0), (I_1,O_1,X_1), \ldots \right\rangle$ the corresponding trace over $\mathsf {AP}$ is $\mathsf {AP} (p) = \langle \mathsf {AP} (I_0,O_0,X_0), \mathsf {AP} (I_1,O_1,X_1), \ldots \rangle$. We lift this definition to sets of traces by defining $\mathsf {APTr} ({\mathcal {S}}) {\mathop {=}\limits ^{\tiny def }}\{\mathsf {AP} (p) \mid p \in {\mathcal {T}} ({\mathcal {S}})\}$.

#### Example 1

Figure 1a shows the formalization of a beverage machine in SMV [46]. In Fig. 1b, we use atomic propositions to reason over the possible values of in and out. This SMV model closely corresponds to an STS: The initial condition predicate $\alpha$ and transition relation $\delta$ are formalized using integer arithmetic as follows:

$$\begin{aligned} \begin{aligned} \alpha {\mathop {=}\limits ^{\tiny def }}&\texttt {out=}\varepsilon \wedge \texttt {wtr=2} \\ \delta {\mathop {=}\limits ^{\tiny def }}&\texttt {in=req} \wedge \texttt {wtr>0} \wedge \texttt {out=coff} \wedge \texttt {wtr'=wtr-1} \vee \\&\texttt {in=req} \wedge \texttt {wtr>0} \wedge \texttt {out=tea} \wedge \texttt {wtr'=wtr-1} \vee \\&\texttt {in=fill} \wedge \texttt {wtr=0} \wedge \lnot \texttt {mut} \wedge \texttt {out=}\varepsilon \wedge \texttt {wtr'=2} \vee \\&\texttt {in=fill} \wedge \texttt {wtr=0} \wedge \texttt {mut} \wedge \texttt {out=}\varepsilon \wedge \texttt {wtr'=1} \vee \\&\texttt {in=}\varepsilon \wedge \texttt {out=}\varepsilon \wedge \texttt {wtr'=wtr} \end{aligned} \end{aligned}$$

The trace $p = \langle (\varepsilon ,\varepsilon ,2),(\texttt {req},\varepsilon ,\texttt {2}), (\texttt {req},\texttt {coff},1)$, $(\varepsilon ,\texttt {tea},0),$ $\ldots \rangle$ is one possible execution of the system (for brevity, variable names are omitted). Examples of atomic propositions for the system are $[\texttt {in=coff}],[\texttt {out=}\varepsilon ],[\texttt {wtr>0}],[\texttt {wtr=0}]$ and the respective atomic proposition trace of *p* is $\mathsf {AP} (p) = \langle \{[\texttt {in=}\varepsilon ],[\texttt {out=}\varepsilon ],[\texttt {wtr>0}]\},$ $\{[\texttt {in=req}],[\texttt {out=}\varepsilon ],[\texttt {wtr>0}]\}, \{[\texttt {in=req}],[\texttt {out=coff}],[\texttt {wtr>0}]\}, \{[\texttt {in=req}],$ $[\texttt {out=tea}],[\texttt {wtr=0}]\} \ldots \rangle$

### HyperLTL

In the following, we provide an overview of the HyperLTL, a logic for hyperproperties, sufficient for understanding the formalization in Sect. 4. For details, we refer the reader to [18]. HyperLTL is defined over atomic proposition traces (see Sect. 2.1) of a fixed STS ${\mathcal {S}} = \langle {\mathcal {I}},{\mathcal {O}},{\mathcal {X}},\alpha ,\delta \rangle$ as defined in Sect. 2.1.

*Syntax.* Let $\mathsf {AP}$ be a set of atomic propositions and let $\pi$ be a *trace variable* from a set ${\mathcal {V}}$ of trace variables. Formulas of HyperLTL are defined by the following grammar:

$$\begin{aligned} \begin{array}{lcccccccccccc} \psi &{} :{:}{=} &{}\exists \pi . \psi &{}|&{} \forall \pi . \psi &{}|&{} \varphi &{}&{}&{}&{}&{}\\ \varphi &{} :{:}{=} &{}a_{\pi } &{}|&{} \lnot \varphi &{}|&{} \varphi \vee \varphi &{}|&{} \mathrm {X} \varphi &{}|&{} \varphi \, \mathrm {U} \varphi \end{array} \end{aligned}$$

Connectives $\exists$ and $\forall$ are universal and existential trace quantifiers, read as “along some traces” and “along all traces”. In our setting, atomic propositions $a \in \mathsf {AP}$ express facts about states or the presence of inputs and outputs. Each atomic proposition is sub-scripted with a trace variable to indicate the trace it is associated with. The Boolean connectives $\wedge$, $\rightarrow$, and $\leftrightarrow$ are defined in terms of $\lnot$ and $\vee$ as usual. Temporal operators $\mathrm {X}$ and $\mathrm {U}$ read *next* and *until*, respectively. Furthermore, we use the standard temporal operators *eventually* $\diamondsuit \varphi {\mathop {=}\limits ^{\tiny def }}\mathsf {true} \,\, \mathrm {U} \varphi$, and *always* $\Box \varphi {\mathop {=}\limits ^{\tiny def }}\lnot \diamondsuit \lnot \varphi$.

*Semantics.* $\varPi \models _{{\mathcal {S}}} \psi$ states that $\psi$ is valid for a given mapping $\varPi : {\mathcal {V}} \rightarrow \mathsf {APTr} ({\mathcal {S}})$ of trace variables to atomic proposition traces. Let $\varPi \left[ \pi \mapsto p \right]$ be as $\varPi$ except that $\pi$ is mapped to *p*. We use $\varPi \left[ i, \infty \right]$ to denote the trace assignment $\varPi '(\pi )=\varPi (\pi )\left[ i, \infty \right]$ for all $\pi$. The validity of a formula is defined as follows:

$$\begin{aligned} \begin{array}{lcccl} \varPi \models _{{\mathcal {S}}} a_{\pi } &{}&{} \text{ iff } &{}&{} a \in \varPi (\pi )[0] \\ \varPi \models _{{\mathcal {S}}} \exists \pi .\psi &{}&{} \text{ iff } &{}&{} \text{ there } \text{ exists } p \in \mathsf {APTr} ({\mathcal {S}}):\\ \varPi \left[ \pi \mapsto p \right] \models _{{\mathcal {S}}} \psi \\ \varPi \models _{{\mathcal {S}}} \forall \pi .\psi &{}&{} \text{ iff } &{}&{} \text{ for } \text{ all } p \in \mathsf {APTr} ({\mathcal {S}}):\\ \varPi \left[ \pi \mapsto p \right] \models _{{\mathcal {S}}} \psi \\ \varPi \models _{{\mathcal {S}}} \lnot \varphi &{}&{} \text{ iff } &{}&{} \varPi \not \models _{{\mathcal {S}}} \varphi \\ \varPi \models _{{\mathcal {S}}} \psi _1 \vee \psi _2 &{}&{} \text{ iff } &{}&{} \varPi \models _{{\mathcal {S}}} \psi _1 \text{ or } \varPi \models _{{\mathcal {S}}} \psi _2 \\ \varPi \models _{{\mathcal {S}}} \mathrm {X} \varphi &{}&{} \text{ iff } &{}&{} \varPi \left[ 1, \infty \right] \models _{{\mathcal {S}}} \varphi \\ \varPi \models _{{\mathcal {S}}} \varphi _1 \, \mathrm {U} \varphi _2 &{}&{} \text{ iff } &{}&{} \text{ there } \text{ exists } i \ge 0:\\ \varPi \left[ i, \infty \right] \models _{{\mathcal {S}}} \varphi _2 \\ &{}&{} &{}&{} \text{ and } \text{ for } \text{ all } 0 \le j < i \text{ we } \text{ have } \\ \varPi \left[ j, \infty \right] \models _{{\mathcal {S}}} \varphi _1 \\ \end{array} \end{aligned}$$

We write ${\mathcal {S}} \models \psi$ if $\varPi \models _{{\mathcal {S}}} \psi$ holds and $\varPi$ is empty. We call $q \in {\mathcal {T}} ({\mathcal {S}})$ a $\pi$-witness of a formula $\exists \pi .\psi$, if $\varPi \left[ \pi \mapsto p \right] \models _{{\mathcal {S}}} \psi$ and $\mathsf {AP} (q) = p$.

### HyperCTL*

HyperCTL* is an extension of HyperLTL described in [18]. We recite the necessary concepts of the logic here and refer the reader to [18] for further details.

*Syntax.* HyperCTL* syntactically is a strict superset of HyperLTL. It allows free mixing temporal operators and path quantifiers. HyperCTL* formulas are defined by the following grammar:

$$\begin{aligned} \varphi :{:}{=} a_{\pi } \mid \lnot \varphi \mid \varphi \vee \varphi \mid \mathrm {X} \varphi \mid \varphi \, \mathrm {U} \varphi \mid \exists \pi \varphi \end{aligned}$$

Further temporal operators, such as $\Box$ and $\lozenge$, are defined as usual. Universal quantification in HyperCTL* is defined via negation and existential quantification: $\forall \pi \varphi {\mathop {=}\limits ^{\tiny def }}\lnot \exists \pi \lnot \varphi$.

*Semantics.* Quantification in HyperCTL* is over paths, which are sequences of system states and atomic proposition pairs, in contrast to HyperLTL where quantification is over sequences of atomic propositions only. In particular, paths assigned to path quantifiers within temporal operators, start in the respective system state currently reasoned over by the temporal operator. For example, a satisfying path assignment for the formula $\exists \pi \Box ((x' = x + 1)_{\pi } \wedge \exists \pi ' (x \ge 0)_{\pi '})$, evaluated over a Kripke structure that initializes some *x* with 0 and increases *x* by an arbitrary amount in each step, assigns to $\pi '$ paths that start in states with $x = 0$, $x = 1$, $x = 2$, etc. In order to disambiguate the notions, we write $\varPi ^*: {\mathcal {V}} \rightarrow ({\mathcal {Y}}\times \mathsf {AP})^{\omega }$ for path assignments, $\models ^*$ for the HyperCTL* modeling relation and $\pi ^*$-witness for witness paths of HyperCTL* formulas. Finally, for ease of presentation, when working with HyperCTL* formulas, we assume that STS have a single initial state and output pair. An arbitrary STS can easily be transformed into this form by introducing a unique initial state variable and output and introducing transitions to all initial states. The formal semantics of HyperCTL* are given as follows:

$$\begin{aligned} \begin{array}{lcccl} \varPi ^* \models ^*_{{\mathcal {S}}} a_{\pi } &{}&{} \text{ iff } &{}&{} a \in \mathsf {AP} (\varPi ^*(\pi )[0]) \\ \varPi ^* \models ^*_{{\mathcal {S}}} \exists \pi .\psi &{}&{} \text{ iff } &{}&{} \text{ there } \text{ exists } y \in ({\mathcal {Y}}\times \mathsf {AP})^{\omega } \text{ such } \text{ that } \\ &{}&{} &{}&{} y[0] = \varPi ^*(\pi )[0] \text{ and } \varPi ^*\left[ \pi \mapsto y \right] \models ^*_{{\mathcal {S}}} \psi \\ \varPi ^* \models ^*_{{\mathcal {S}}} \lnot \varphi &{}&{} \text{ iff } &{}&{} \varPi ^* \not \models ^*_{{\mathcal {S}}} \varphi \\ \varPi ^* \models ^*_{{\mathcal {S}}} \mathrm {X} \varphi &{}&{} \text{ iff } &{}&{} \varPi ^*\left[ 1, \infty \right] \models ^*_{{\mathcal {S}}} \varphi \\ \varPi ^* \models ^*_{{\mathcal {S}}} \varphi _1 \, \mathrm {U} \varphi _2 &{}&{} \text{ iff } &{}&{} \text{ there } \text{ exists } i \ge 0: \varPi ^*\left[ i, \infty \right] \models ^*_{{\mathcal {S}}} \varphi _2 \\ &{}&{} &{}&{} \text{ and } \text{ for } \text{ all } 0 \le j < i \text{ we } \text{ have } \varPi ^*\left[ j, \infty \right] \models ^*_{{\mathcal {S}}} \varphi _1 \\ \end{array} \end{aligned}$$

## Killing mutants

In this section, we introduce mutants, linear and locally adaptive tests, and the notions of potential and definite killing. Furthermore, we discuss how to represent an STS and its corresponding mutant as a single STS, which can then be model checked to determine killability.

### Mutants

Mutants are variations of a model ${\mathcal {S}}$ obtained by applying small modifications to the syntactic representation of ${\mathcal {S}}$. A mutant of an STS ${\mathcal {S}} = \langle {\mathcal {I}},{\mathcal {O}},{\mathcal {X}},\alpha ,\delta \rangle$ (the *original model*) is an STS ${\mathcal {S}} ^m = \langle {\mathcal {I}},{\mathcal {O}},{\mathcal {X}},\alpha ^m,\delta ^m \rangle$ with equal sets of input, output, and state variables as ${\mathcal {S}}$ but a deviating initial predicate and/or transition relation. We assume that ${\mathcal {S}} ^m$ is equally input-enabled as ${\mathcal {S}}$, that is ${\mathcal {T}} ({\mathcal {S}} ^m)\vert _{{{\mathcal {I}}}} = {\mathcal {T}} ({\mathcal {S}})\vert _{{{\mathcal {I}}}}$, i.e., the mutant and model accept the same sequences of inputs. In practice, this can easily be achieved by using self-loops with empty output to ignore unspecified inputs. We use standard mutation operators, such as disabling transitions, and replacing operators. The mutation operators used in our experiments are presented in Sect. 6 and in [7]. We combine an original model represented by ${\mathcal {S}}$ and a mutant ${\mathcal {S}} ^m$ into a *conditional mutant* ${\mathcal {S}} ^{c(m)}$, in order to perform mutation analysis via model checking the combined model.

The conditional mutant is defined as ${\mathcal {S}} ^{c(m)}~{\mathop {=}\limits ^{\tiny def }}~\langle {\mathcal {I}},{\mathcal {O}},{\mathcal {X}} ~\cup ~\{\mathrm {mut}\}, \alpha ^{c(m)},\delta ^{c(m)}\rangle$, where $\mathrm {mut}$ is a fresh Boolean variable used to distinguish states of the original and the mutated STS. Suppose ${\mathcal {S}} ^m$ replaces a sub-formula $\delta _0$ of $\delta$ by $\delta _0^m$, then the transition relation predicate of the conditional mutant $\delta ^{c(m)}$ is obtained by replacing $\delta _0$ in $\delta$ by $(\mathrm {mut}\wedge \delta _0^m) \vee (\lnot \mathrm {mut}\wedge \delta _0)$. We fix the value of $\mathrm {mut}$ in transitions adding the conjunct $\mathrm {mut}\leftrightarrow \mathrm {mut}'$ to $\delta$. The initial conditions predicate of the conditional mutant is defined similarly.

Consequently, for a trace $p \in {\mathcal {T}} ({\mathcal {S}} ^{c(m)})$ it holds that if $p|_{\{\mathrm {mut}\}}=\{\bot \}^{\omega }$ then $p\vert _{{{\mathcal {I}} \cup {\mathcal {O}} \cup {\mathcal {X}}}} \in {\mathcal {T}} ({\mathcal {S}})$, and if $p|_{\{\mathrm {mut}\}}=\{\top \}^{\omega }$ then $p\vert _{{{\mathcal {I}} \cup {\mathcal {O}} \cup {\mathcal {X}}}} \in {\mathcal {T}} ({\mathcal {S}} ^m)$. Formally, ${\mathcal {S}} ^{c(m)}$ is non-deterministic, since $\mathrm {mut}$ is chosen non-deterministically in the initial state. However, we only refer to ${\mathcal {S}} ^{c(m)}$ as non-deterministic if either ${\mathcal {S}}$ or ${\mathcal {S}} ^m$ is non-deterministic, as $\mathrm {mut}$ is fixed in the hypertproperties presented in Sect. 4.

Example 1 and Fig. 1a show a conditional mutant as an STS and in SMV.

### Killing

Killing a mutant amounts to finding inputs for which the mutant produces outputs that deviate from the original model. In a reactive, model-based setting, killing has been formalized using conformance relations [52], for example in [4, 27], where an implementation *conforms* to its specification if all its input/output sequences are part of/allowed by the specification.

In model-based testing, the model takes the role of the specification and is assumed to be correct by design. The implementation is treated as black box, and therefore mutants of the specification serve as its proxy. Tests that demonstrate non-conformance between the model and its mutant can be used to verify whether a system under test is an implementation of the specification or contains the bug reflected in the mutant.

What exactly constitutes demonstration of non-conformance is system dependent. In particular, it depends on whether the model from which tests are created is deterministic or non-deterministic. In the following paragraphs, we discuss these differences. We start by defining tests and their verdicts.

The simplest definition of a test for a reactive system is a sequence of inputs and outputs, which is typically called a linear test. The execution of a linear test on a system under test *fails* if the sequence of inputs of the test triggers a sequence of outputs that deviates from those predicted by the test and *passes* otherwise. Formally, linear tests are defined as follows:

#### Definition 1

(Linear Test) A *linear test* *t* of *length* *n* for ${\mathcal {S}}$ comprises inputs $t\vert _{{{\mathcal {I}}}}$ and outputs $t\vert _{{{\mathcal {O}}}}$ of length *n*, such that there exists a trace $p \in {\mathcal {T}} ({\mathcal {S}})$ with $p[0,n]\vert _{{{\mathcal {I}}}}~=~t\vert _{{{\mathcal {I}}}}$ and $p[0,n]\vert _{{{\mathcal {O}}}}~=~t\vert _{{{\mathcal {O}}}}$.

**Fig. 2:**
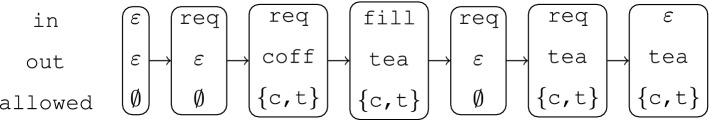
Definitely killing locally adaptive test (c = coff, t = tea)

Linear tests can be problematic for non-deterministic models. A conformant implementation of a non-deterministic model may resolve some non-deterministic choice of the model in a different order to a given linear test. As a result, the implementation delivers an output that is different to the output of the test and the test fails, even though the delivered output is allowed by the model. To remedy this situation tests can be extended with information on multiple non-deterministic choices. This can either be done by extending a linear test to a fully adaptive tree that branches out in every non-deterministic choice, or by adding sets of allowed outputs to the test. We discuss here the latter variant.

A locally adaptive test is a sequence of inputs, outputs, and locally allowed outputs, where an output *O* is locally allowed for some state and input *I*, if there is a successor state corresponding to input *I* that has output *O*. The execution of a locally adaptive test on a system under test *passes* if its sequence of inputs triggers its exact sequence of outputs. The execution is *inconclusive* as soon as an allowed output is given by the system under test that is different to the test’s output. The execution *fails* as soon as an output that is not allowed is given by the system under test. Note that allowed output information is local in the sense that it follows some state. A stronger notion would be globally allowed output that follows some sequence of inputs and outputs. However, expressing this notion is beyond the capabilities of current logics for hyperproperties and thus is not discussed further.

In order to express locally adaptive tests, we extend symbolic transition systems with indicator variables for allowed outputs. Furthermore, in Sect. 4, we discuss hyperproperties expressing this property.

Let $\mathop {Out}$ be the set of all outputs. Remember that an output *O* is a mapping of output variables ${\mathcal {O}}$ to a range of output values. Therefore, $\mathop {Out}$ is a set of mappings. We define locally allowed output indicators as the set of fresh Boolean variables ${\mathcal {A}} {:}{=} \{a[O] \mid O \in \mathop {Out}\}$ as a subset of state variables ${\mathcal {X}}$ that are not used in the initial state or transition predicate.

#### Definition 2

(Locally adaptive test) A *locally adaptive test* *t* of *length* *n* for ${\mathcal {S}}$ comprises inputs $t\vert _{{{\mathcal {I}}}}$, outputs $t\vert _{{{\mathcal {O}}}}$, and allowed outputs $t\vert _{{{\mathcal {A}}}}$ of length *n*, such that there exists a trace $p \in {\mathcal {T}} ({\mathcal {S}})$ with $p[0,n]\vert _{{{\mathcal {I}}}}~=~t\vert _{{{\mathcal {I}}}}$, $p[0,n]\vert _{{{\mathcal {O}}}}~=~t\vert _{{{\mathcal {O}}}}$ and such that for every $j \in [0,n]$ and every $O \in \mathop {Out}$ it is the case that *t*[*j*] at *a*[*O*] evaluates to $\top$ if and only if there exists a trace $p' \in {\mathcal {T}} ({\mathcal {S}})$ with $p'[0,j-1]~=~t[0,j-1]$, $p'[j]\vert _{{{\mathcal {I}}}}~=~t[j]\vert _{{{\mathcal {I}}}}$, and $p'[j]\vert _{{{\mathcal {O}}}}~=~O$.

#### Example 2

Consider again the linear test presented in Fig. 1c. Figure 2 shows the locally adaptive version of that test. Note that the allowed part is represented as the set of allowed outputs.

Non-determinism does not only need to be taken into account during test execution, but already during test creation. For non-deterministic models, we differentiate between two different strengths of killing. We say that a mutant can be *potentially killed* if there exist inputs for which the mutant’s outputs deviate from the original model given an appropriate choice of non-deterministic initial states and transitions. In practice, executing a test that potentially kills a mutant on a faulty implementation that exhibits non-determinism (e.g., a multi-threaded program) may fail to demonstrate non-conformance (unless the non-determinism can be controlled). In case non-determinism can not be controlled and the system under test exactly implements the mutant, then a potentially killing test passes on some executions and fails on others. Such tests are sometimes referred to as a flaky test, which are generally undesirable. Since non-determinism can not always be controlled in practice and system under tests can be non-deterministic, we provide a stronger notion of killing. A mutant can be *definitely killed* if there exists a sequence of inputs for which the behaviors of the mutant and the original model deviate independently of how non-determinism is resolved.

Note potential and definite killability are orthogonal to the well known notions of weak and strong killing, which capture different degrees of observability. Formally, we define potential and definite killability as follows:

#### Definition 3

(Potentially killable) ${\mathcal {S}} ^m$ is *potentially killable* if

$$\begin{aligned} {\mathcal {T}} ({\mathcal {S}} ^m)\vert _{{{\mathcal {I}} \cup {\mathcal {O}}}} \nsubseteq {\mathcal {T}} ({\mathcal {S}})\vert _{{{\mathcal {I}} \cup {\mathcal {O}}}} \end{aligned}$$

Test *t* (locally adaptive or linear) for ${\mathcal {S}}$ of length *n* *potentially kills* ${\mathcal {S}} ^m$ if

$$\begin{aligned}&\{q[0,n] \mid q \in {\mathcal {T}} ({\mathcal {S}} ^m) \wedge q[0,n]\vert _{{{\mathcal {I}}}} = t\vert _{{{\mathcal {I}}}}\}\vert _{{{\mathcal {I}} \cup {\mathcal {O}}}} \\&\quad \nsubseteq \{p[0,n] \mid p \in {\mathcal {T}} ({\mathcal {S}})\}\vert _{{{\mathcal {I}} \cup {\mathcal {O}}}}. \end{aligned}$$

#### Definition 4

(Definitely killable) ${\mathcal {S}} ^m$ is *definitely killable* if there is a sequence of inputs $\vec {I} \in {\mathcal {T}} ({\mathcal {S}})\vert _{{{\mathcal {I}}}}$, such that

$$\begin{aligned} \{q \in {\mathcal {T}} ({\mathcal {S}} ^m) \mid q\vert _{{{\mathcal {I}}}} = \vec {I}\} \vert _{{{\mathcal {O}}}} \cap \{p \in {\mathcal {T}} ({\mathcal {S}}) \mid p\vert _{{{\mathcal {I}}}} = \vec {I}\}\vert _{{{\mathcal {O}}}} = \emptyset \end{aligned}$$

Test *t* (locally adaptive or linear) for ${\mathcal {S}}$ of length *n* *definitely kills* ${\mathcal {S}} ^m$ if

$$\begin{aligned} \begin{aligned}&\{q[0,n] \mid q \in {\mathcal {T}} ({\mathcal {S}} ^m) \wedge q[0,n]\vert _{{{\mathcal {I}}}} = t\vert _{{{\mathcal {I}}}}\}\vert _{{{\mathcal {O}}}} \cap \\&\{p[0,n] \mid p \in {\mathcal {T}} ({\mathcal {S}}) \wedge p[0,n]\vert _{{{\mathcal {I}}}} = t\vert _{{{\mathcal {I}}}}\}\vert _{{{\mathcal {O}}}} = \emptyset \end{aligned} \end{aligned}$$

#### Definition 5

(Equivalent Mutant) ${\mathcal {S}} ^m$ is *equivalent* iff ${\mathcal {S}} ^m$ is not potentially killable.

The following proposition relates definite and potential killabilty:

#### Proposition 1

If ${\mathcal {S}} ^m$ is definitely killable then ${\mathcal {S}} ^m$ is potentially killable. If ${\mathcal {S}} ^m$ is deterministic then: ${\mathcal {S}} ^m$ is potentially killable iff ${\mathcal {S}} ^m$ is definitely killable.

#### Proof

Let ${\mathcal {S}} ^m$ be definitely killable. Then there is a trace $q \in {\mathcal {T}} ({\mathcal {S}} ^m)$, such that there is no trace $p \in {\mathcal {T}} ({\mathcal {S}})$ with $q\vert _{{{\mathcal {I}} \cup {\mathcal {O}}}} = p\vert _{{{\mathcal {I}} \cup {\mathcal {O}}}}$, which implies ${\mathcal {T}} ({\mathcal {S}} ^m)\vert _{{{\mathcal {I}} \cup {\mathcal {O}}}} \nsubseteq {\mathcal {T}} ({\mathcal {S}})\vert _{{{\mathcal {I}} \cup {\mathcal {O}}}}$.

Let ${\mathcal {S}}$ be deterministic and ${\mathcal {S}} ^m$ be potentially killable. From the definition of determinism it follows that for traces $q,q' \in {\mathcal {T}} ({\mathcal {S}} ^m)$ with $q\vert _{{{\mathcal {I}}}} = q'\vert _{{{\mathcal {I}}}}$ it is the case that $q = q'$. In other words, for every sequence of inputs $\vec {I}$ it is the case that $|\{q \in {\mathcal {T}} ({\mathcal {S}} ^m) \mid q\vert _{{{\mathcal {I}}}} = \vec {I}\}\vert _{{{\mathcal {O}}}}| \le 1$. From potential killability (i.e. ${\mathcal {T}} ({\mathcal {S}} ^m)\vert _{{{\mathcal {I}} \cup {\mathcal {O}}}} \nsubseteq {\mathcal {T}} ({\mathcal {S}})\vert _{{{\mathcal {I}} \cup {\mathcal {O}}}}$) it follows that there exists $q \in {\mathcal {T}} ({\mathcal {S}} ^m)$, such that $q\vert _{{{\mathcal {O}}}} \notin \{p \in {\mathcal {T}} ({\mathcal {S}} ^m) \mid p\vert _{{{\mathcal {I}}}} = q\vert _{{{\mathcal {I}}}}\}\vert _{{{\mathcal {O}}}}$. Since the set of traces in the mutant sharing inputs with *q* is a singleton, it is the case that $\{q' \in {\mathcal {T}} ({\mathcal {S}} ^m) \mid q'\vert _{{{\mathcal {I}}}} = q\vert _{{{\mathcal {I}}}}\}\vert _{{{\mathcal {O}}}} \cap \{p \in {\mathcal {T}} ({\mathcal {S}}) \mid p\vert _{{{\mathcal {I}}}} = q\vert _{{{\mathcal {I}}}}\}\vert _{{{\mathcal {O}}}} = \emptyset$. Therefore, *q* is a witness to ${\mathcal {S}} ^m$ being definitely killable. $\square$

In summary, definite killability is stronger than potential killabilty, though for deterministic mutants, the two notions coincide. Therefore, for deterministic mutants, we simply speak of killing and tests that kill. The following example shows a definitely killable mutant, a mutant that is only potentially killable, and an equivalent mutant.

#### Example 3

The mutant in Fig. 1a, is definitely killable, since we can force the system into a state in which both possible outputs of the original system (coff, tea) differ from the only possible output of the mutant ($\varepsilon$).

Consider a mutant that introduces non-determinism by replacing line 10 with **if**(in=fill&wtr=0):(mut?{1,2}:2), indicating that the machine is filled with either 1 or 2 units of water. This mutant is potentially but not definitely killable, as only one of the non-deterministic choices leads to a deviation of outputs.

Finally, consider a mutant that replaces line 7 with **if**(in=req&wtr>0):(mut ? coff:{coff,tea}) and removes the mut branch of line 10, yielding a machine that always creates coffee. Every implementation of this mutant is also correct with respect to the original model. Hence, we consider the mutant equivalent, even though the original model, unlike the mutant, can output tea.

## Killing with hyperproperties

In this section, we provide a formalization of potential and definite killability in terms of HyperLTL, prove the correctness of our formalization with respect to Sect. 3, and explain how tests can be extracted by model checking the HyperLTL properties. Furthermore, we present an encoding of locally adaptive tests in HyperCTL*.

All HyperLTL formulas presented in this section depend on inputs and outputs of the model, but are model-agnostic otherwise. The idea of all presented formulas is to discriminate between traces of the original model ($\Box \lnot \mathrm {mut}_{\pi }$) and traces of the mutant ($\Box \mathrm {mut}_{\pi }$). Furthermore, we quantify over pairs $(\pi ,\pi ')$ of traces with globally equal inputs $(\Box (I_{\pi } \leftrightarrow I_{\pi '}))$ and express that such pairs will eventually have different outputs $(\lozenge (O_{\pi } \not \leftrightarrow O_{\pi '}))$, where for ease of presentation, we abbreviate $\bigwedge _{i \in \mathsf {AP} _{{\mathcal {I}}}} (i_{\pi } \leftrightarrow i_{\pi '})$ by $I_{\pi } \leftrightarrow I_{\pi '}$ and $\bigvee _{o \in \mathsf {AP} _{{\mathcal {O}}}} \lnot ( o_{\pi } \leftrightarrow o_{\pi '})$ by $O_{\pi } \not \leftrightarrow O_{\pi '}$. We start by showing some general properties used throughout the following HyperLTL formalizations of killability.

### Lemma 1

Let $\varPi$ be a trace assignment, ${\mathcal {S}} ^{c(m)}$ a conditional mutant, and let *p*, *q* be sequences of system states of ${\mathcal {S}} ^{c(m)}$ with $\mathsf {AP} (p) = \varPi (\pi )$, $\mathsf {AP} (q) = \varPi (\pi ')$.

1. $$\varPi \models _{{\mathcal {S}} ^{c(m)}} \Box \lnot \mathrm {mut}_{\pi }$$ then $$p\vert _{{{\mathcal {I}} \cup {\mathcal {O}} \cup {\mathcal {X}}}} \in {\mathcal {T}} ({\mathcal {S}})$$
2. $$\varPi \models _{{\mathcal {S}} ^{c(m)}} \Box \mathrm {mut}_{\pi }$$ then $$p\vert _{{{\mathcal {I}} \cup {\mathcal {O}} \cup {\mathcal {X}}}} \in {\mathcal {T}} ({\mathcal {S}} ^m)$$
3. $$\varPi \models _{{\mathcal {S}} ^{c(m)}} \Box \big (\bigwedge _{i \in \mathsf {AP} _{{\mathcal {I}}}} (i_{\pi } \leftrightarrow i_{\pi '})\big )$$ then $$p\vert _{{{\mathcal {I}}}} = q\vert _{{{\mathcal {I}}}}$$
4. $$\varPi \models _{{\mathcal {S}} ^{c(m)}} \lozenge \big (\bigvee _{o \in \mathsf {AP} _{{\mathcal {O}}}} \lnot ( o_{\pi } \leftrightarrow o_{\pi '})\big )$$ then $$p\vert _{{{\mathcal {O}}}} \ne q\vert _{{{\mathcal {O}}}}$$

### Proof

The first two statements follow directly from the definition of conditional mutants. The latter two statements follow directly from the fact that $\mathsf {AP} _{{\mathcal {I}}},\mathsf {AP} _{{\mathcal {O}}}$ uniquely characterize inputs and outputs. $\square$

### Deterministic model and mutant

To show killability (potential and definite) of a deterministic mutant for a deterministic model, one needs to find a trace of the model ($\exists \pi$) such that the trace of the mutant with the same inputs ($\exists \pi '$) eventually diverges in outputs, which is formalized via the hyperproperty $\phi _1$ as follows:

$$\begin{aligned} \phi _1({\mathcal {I}},{\mathcal {O}}) {:}{=} \exists \pi \exists \pi ' \Box \big (\lnot \mathrm {mut}_{\pi } \wedge \mathrm {mut}_{\pi '} \wedge (I_{\pi } \leftrightarrow I_{\pi '})\big ) \wedge \lozenge \big (O_{\pi } \not \leftrightarrow O_{\pi '}\big ) \end{aligned}$$

#### Proposition 2

For a deterministic model ${\mathcal {S}}$ and mutant ${\mathcal {S}} ^m$, it holds that

$$\begin{aligned} {\mathcal {S}} ^{c(m)} \models \phi _1({\mathcal {I}},{\mathcal {O}}) \text { iff } {\mathcal {S}} ^m \text { is killable}. \end{aligned}$$

If *p* is a $\pi$-witness for ${\mathcal {S}} ^{c(m)} \models \phi _1({\mathcal {I}},{\mathcal {O}})$, then there exists $n \in {\mathbb {N}}$ such that test $t {\mathop {=}\limits ^{\tiny def }}p[0,n]\vert _{{{\mathcal {I}} \cup {\mathcal {O}}}}$ kills ${\mathcal {S}} ^m$.

#### Proof

We show that ${\mathcal {S}} ^m$ is potentially killable *iff* ${\mathcal {S}} ^{c(m)} \models \phi _1({\mathcal {I}},{\mathcal {O}})$. This suffices, since by Lemma 1 and due to the fact that ${\mathcal {S}} ^m$ is deterministic, ${\mathcal {S}} ^m$ is definitely killable *iff* ${\mathcal {S}} ^m$ is potentially killable.

Assume ${\mathcal {S}} ^m$ is potentially killable. Let $q \in {\mathcal {T}} ({\mathcal {S}} ^m)$, such that $q\vert _{{{\mathcal {I}} \cup {\mathcal {O}}}} \notin {\mathcal {T}} ({\mathcal {S}})\vert _{{{\mathcal {I}} \cup {\mathcal {O}}}}$. Since ${\mathcal {S}} ^m$ is equally input-enabled, there exists a trace $p \in {\mathcal {T}} ({\mathcal {S}})$, such that $p\vert _{{{\mathcal {I}}}} = q\vert _{{{\mathcal {I}}}}$. Clearly, $p\vert _{{{\mathcal {O}}}} \ne q\vert _{{{\mathcal {O}}}}$. Therefore, *p* and *q* are satisfying assignments for $\phi _1({\mathcal {I}},{\mathcal {O}})$ and $\pi$, $\pi '$ respectively.

Conversely, assume ${\mathcal {S}} ^{c(m)} \models \phi _1({\mathcal {I}},{\mathcal {O}})$. Let *p*, *q* be a $\pi ,\pi '$-witnesses of $\phi _1({\mathcal {I}},{\mathcal {O}})$. From Lemma 1, we immediately get $p\vert _{{{\mathcal {I}}}} = q\vert _{{{\mathcal {I}}}}$, and $p\vert _{{{\mathcal {O}}}} \ne q\vert _{{{\mathcal {O}}}}$ , which shows ${\mathcal {T}} ({\mathcal {S}} ^m)\vert _{{{\mathcal {I}} \cup {\mathcal {O}}}} \nsubseteq {\mathcal {T}} ({\mathcal {S}})\vert _{{{\mathcal {I}} \cup {\mathcal {O}}}}$.

Since $p\vert _{{{\mathcal {O}}}} \ne q\vert _{{{\mathcal {O}}}}$, there exists an $n \in {\mathbb {N}}$ such that $p[0,n-1]\vert _{{{\mathcal {O}}}} = q[0,n-1]\vert _{{{\mathcal {O}}}}$ and $p[n]\vert _{{{\mathcal {O}}}} \ne q[n]\vert _{{{\mathcal {O}}}}$. Clearly, the test $t{\mathop {=}\limits ^{\tiny def }}p[0,n]\vert _{{{\mathcal {I}} \cup {\mathcal {O}}}}$ kills ${\mathcal {S}} ^m$. $\square$

### Non-deterministic model and mutant

For potential killability of non-deterministic models and mutants, we need to find a trace of the mutant ($\exists \pi$) such that all traces of the model with the same inputs ($\forall \pi '$) eventually diverge in outputs, which is formalized via the hyperproperty $\phi _2$ as follows:

$$\begin{aligned} \phi _2({\mathcal {I}},{\mathcal {O}}) {:}{=} \exists \pi \forall \pi ' \Box \mathrm {mut}_{\pi } \wedge&\Big (\Box \big (\lnot \mathrm {mut}_{\pi '} \wedge (I_{\pi } \leftrightarrow I_{\pi '})\big ) \rightarrow \lozenge \big (O_{\pi } \not \leftrightarrow O_{\pi '}\big )\Big ) \end{aligned}$$

#### Proposition 3

For non-deterministic ${\mathcal {S}}$ and ${\mathcal {S}} ^m$, it holds that

$$\begin{aligned} {\mathcal {S}} ^{c(m)} \models \phi _2({\mathcal {I}},{\mathcal {O}}) \text { iff } {\mathcal {S}} ^m \text { is potentially killable.} \end{aligned}$$

If *q* is a $\pi$-witness for ${\mathcal {S}} ^{c(m)} \models \phi _2({\mathcal {I}},{\mathcal {O}})$, then for any trace $p \in {\mathcal {T}} ({\mathcal {S}})$ with $q\vert _{{{\mathcal {I}}}} = p\vert _{{{\mathcal {I}}}}$ there is $n \in {\mathbb {N}}$ such that the test $t {\mathop {=}\limits ^{\tiny def }}p[0,n]\vert _{{{\mathcal {I}} \cup {\mathcal {O}}}}$ potentially kills ${\mathcal {S}} ^m$.

#### Proof

Assume that ${\mathcal {S}} ^m$ is potentially killable. That is, there is a trace $q \in {\mathcal {T}} ({\mathcal {S}} ^m)$, such that there is no trace $p \in {\mathcal {T}} ({\mathcal {S}})$ with $q\vert _{{{\mathcal {I}} \cup {\mathcal {O}}}} = p\vert _{{{\mathcal {I}} \cup {\mathcal {O}}}}$. Any trace assignment that maps $\pi$ to *q* satisfies $\phi _2({\mathcal {I}},{\mathcal {O}})$, since that assignment either violates the antecedent by mapping a trace $p \in {\mathcal {T}} ({\mathcal {S}})$ with different inputs than *q* to $\pi '$, or it violates the consequent by mapping a trace $p \in {\mathcal {T}} ({\mathcal {S}})$ to $\pi '$ with inputs $q\vert _{{{\mathcal {I}}}}$ and outputs that can only be different to $q\vert _{{{\mathcal {O}}}}$.

Conversely, assume ${\mathcal {S}} ^{c(m)} \models \phi _2({\mathcal {I}},{\mathcal {O}})$. Let *p* be a $\pi$-witness and *q* be a $\pi '$-witness for which the antecedent of the implication is satisfied, which is in fact satisfiable, since ${\mathcal {S}} ^m$ is equally input-enabled. Clearly, *p* is a $\pi$-witness for $\Box \mathrm {mut}_{\pi }$ and since *q* is chosen such that it satisfies the antecedent, *q* is a $\pi '$-witness for $\Box \lnot \mathrm {mut}_{\pi '}$. Thus, from Lemma 1, we get $p\vert _{{{\mathcal {I}} \cup {\mathcal {O}} \cup {\mathcal {X}}}} \in {\mathcal {T}} ({\mathcal {S}})$, $q\vert _{{{\mathcal {I}} \cup {\mathcal {O}} \cup {\mathcal {X}}}} \in {\mathcal {T}} ({\mathcal {S}} ^m)$, and $q\vert _{{{\mathcal {I}}}} = p\vert _{{{\mathcal {I}}}}$. Since $\varPi [\pi \mapsto p, \pi ' \mapsto q]$ satisfies the antecedent of the implication and the whole formula, the trace assignment also satisfies the consequent of the implication , i.e., $q\vert _{{{\mathcal {O}}}} \ne p\vert _{{{\mathcal {O}}}}$ (Lemma 1). Since *q* was chosen arbitrary (besides satisfying the antecedent), we can conclude $p\vert _{{{\mathcal {I}} \cup {\mathcal {O}}}} \notin {\mathcal {T}} ({\mathcal {S}} ^m)\vert _{{{\mathcal {I}} \cup {\mathcal {O}}}}$, i.e., ${\mathcal {S}} ^m$ is potentially killable.

Let $q \in {\mathcal {T}} ({\mathcal {S}} ^m)$ be a $\pi$-witness to ${\mathcal {S}} ^{c(m)} \models \phi _2({\mathcal {I}},{\mathcal {O}})$ and let $p \in {\mathcal {T}} ({\mathcal {S}})$ be any trace with $p\vert _{{{\mathcal {I}}}} = q\vert _{{{\mathcal {I}}}}$, which exists since ${\mathcal {S}} ^m$ is equally input-enabled. Clearly, there exists an $n \in {\mathbb {N}}$ such that $q[0,n-1]\vert _{{{\mathcal {O}}}} = p[0,n-1]\vert _{{{\mathcal {O}}}}$ and $q[n]\vert _{{{\mathcal {O}}}} \ne p[n]\vert _{{{\mathcal {O}}}}$. Therefore, the test $t {\mathop {=}\limits ^{\tiny def }}p[0,n]\vert _{{{\mathcal {I}} \cup {\mathcal {O}}}}$ potentially kills ${\mathcal {S}} ^m$. $\square$

For definite killability one needs to find a sequence of inputs of the model ($\exists \pi$) and compare all non-deterministic outcomes of the model ($\forall \pi ''$) to all non-deterministic outcomes of the mutant ($\forall \pi '$) for these inputs, which is formalized via the hyperproperty $\phi _3$ as follows:

$$\begin{aligned}&\phi _3({\mathcal {I}},{\mathcal {O}}) {\mathop {=}\limits ^{\tiny def }}\\&\exists \pi \forall \pi ' \forall \pi '' \Box \lnot \mathrm {mut}_{\pi } \wedge \Big (\Box \big (\mathrm {mut}_{\pi '} \wedge \lnot \mathrm {mut}_{\pi ''} \wedge (I_{\pi } \leftrightarrow I_{\pi '}) \wedge (I_{\pi } \leftrightarrow I_{\pi ''})\big ) \rightarrow \\&\quad \quad \quad \quad \quad \quad \quad \quad \quad \quad \quad \quad \quad \quad \quad \quad \quad \quad \quad \quad \quad \quad \quad \quad \quad \quad \quad \quad \quad \quad \lozenge \big (O_{\pi '} \not \leftrightarrow O_{\pi ''}\big )\Big ) \end{aligned}$$

In Fig. 1b, we present an instance of $\phi _3$ for our running example.

#### Proposition 4

For non-deterministic ${\mathcal {S}}$ and ${\mathcal {S}} ^m$, it holds that

$$\begin{aligned} {\mathcal {S}} ^{c(m)} \models \phi _3({\mathcal {I}},{\mathcal {O}}) \text { iff } {\mathcal {S}} ^m \text { is definitely killable.} \end{aligned}$$

If ${\mathcal {S}} ^{m}$ is finite and *p* is a $\pi$-witness for ${\mathcal {S}} ^{c(m)} \models \phi _3({\mathcal {I}},{\mathcal {O}})$, then there exists $n \in {\mathbb {N}}$, such that the test $t {\mathop {=}\limits ^{\tiny def }}p[0,n]\vert _{{{\mathcal {I}} \cup {\mathcal {O}}}}$ definitely kills ${\mathcal {S}} ^m$.

#### Proof

Let ${\mathcal {S}} ^m$ be definitely killable, which implies that there is a sequence of inputs $\vec {I} \in {\mathcal {T}} ({\mathcal {S}})\vert _{{{\mathcal {I}}}}$, such that for $P_{\vec {I}} {\mathop {=}\limits ^{\tiny def }}\{p \in {\mathcal {T}} ({\mathcal {S}}) \mid p\vert _{{{\mathcal {I}}}} = \vec {I}\}$ and $Q_{\vec {I}} {\mathop {=}\limits ^{\tiny def }}\{q \in {\mathcal {T}} ({\mathcal {S}} ^m) \mid q\vert _{{{\mathcal {I}}}} = \vec {I}\}$ it is the case that $P_{\vec {I}}\vert _{{{\mathcal {O}}}} \cap Q_{\vec {I}}\vert _{{{\mathcal {O}}}} = \emptyset$. Since $\vec {I}$ is the input sequence of a trace of ${\mathcal {S}}$, we have that $P_{\vec {I}} \ne \emptyset$. Since ${\mathcal {S}} ^m$ is equally input-enabled, we have $Q_{\vec {I}} \ne \emptyset$. We show that any $p \in P_{\vec {I}}$ is a $\pi$-witness to ${\mathcal {S}} ^{c(m)} \models \phi _3({\mathcal {I}},{\mathcal {O}})$. Let $q' \in {\mathcal {T}} ({\mathcal {S}} ^m)$ and $p'' \in {\mathcal {T}} ({\mathcal {S}})$ be arbitrary traces, consider a trace assignment that maps $\pi$ to *p*, $\pi '$ to $q'$ and $\pi ''$ to $p''$ and assume that it satisfies the antecedent (which is satisfiable, due to $P_{\vec {I}} \ne \emptyset$ and $Q_{\vec {I}} \ne \emptyset$). That is, $q' \in Q_{\vec {I}}$ and $p'' \in P_{\vec {I}}$. Since $P_{\vec {I}}\vert _{{{\mathcal {O}}}} \cap Q_{\vec {I}}\vert _{{{\mathcal {O}}}} = \emptyset$, it must be the case that $q'\vert _{{{\mathcal {O}}}} \ne p''\vert _{{{\mathcal {O}}}}$. Since $q'$ and $p''$ were chosen arbitrarily, any trace assignment that maps *p* to $\pi$ satisfies the formula, i.e.,, ${\mathcal {S}} ^{c(m)} \models \phi _3({\mathcal {I}},{\mathcal {O}})$.

Conversely, assume ${\mathcal {S}} ^{c(m)} \models \phi _3({\mathcal {I}},{\mathcal {O}})$. Let *p* be a $\pi$-witness, and let $q'$ and $p''$ be $\pi '$ and $\pi ''$-witnesses for which the antecedent is satisfied, which is in fact satisfiable, since ${\mathcal {S}} ^m$ is equally input-enabled. Clearly, *p* is a $\pi$-witness for $\Box \lnot \mathrm {mut}_{\pi }$ and since $q'$ and $p''$ were chosen such that they satisfy the antecedent, $q'$ is a $\pi '$-witness for $\Box \mathrm {mut}_{\pi '}$ and *p* is a $\pi ''$-witness for $\Box \lnot \mathrm {mut}_{\pi ''}$. Thus, from Lemma 1, we get $p\vert _{{{\mathcal {I}} \cup {\mathcal {O}} \cup {\mathcal {X}}}},p''\vert _{{{\mathcal {I}} \cup {\mathcal {O}} \cup {\mathcal {X}}}} \in {\mathcal {T}} ({\mathcal {S}})$, $q'\vert _{{{\mathcal {I}} \cup {\mathcal {O}} \cup {\mathcal {X}}}} \in {\mathcal {T}} ({\mathcal {S}} ^m)$, and $p\vert _{{{\mathcal {I}}}} = q'\vert _{{{\mathcal {I}}}} = p''\vert _{{{\mathcal {I}}}}$.

Since the $\varPi [\pi \mapsto p, \pi ' \mapsto q',\pi '' \mapsto p'']$ satisfies the whole formula and the antecedent, the trace assignment must also satisfy the consequent. That is, it must be the case that $q'\vert _{{{\mathcal {O}}}} \ne p''\vert _{{{\mathcal {O}}}}$ (Lemma 1). Since $q'$ and $p''$ were chosen arbitrarily (besides satisfying the antecedent), we have shown $\{q \in {\mathcal {T}} ({\mathcal {S}} ^m) \mid q\vert _{{{\mathcal {I}}}} = p\vert _{{{\mathcal {I}}}}\}\vert _{{{\mathcal {O}}}} \cap \{p'' \in {\mathcal {T}} ({\mathcal {S}}) \mid p''\vert _{{{\mathcal {I}}}} = p\vert _{{{\mathcal {I}}}}\}\vert _{{{\mathcal {O}}}} = \emptyset$, i.e. $\vec {I} {\mathop {=}\limits ^{\tiny def }}p\vert _{{{\mathcal {I}}}}$ is the input sequence showing that ${\mathcal {S}} ^m$ is definitely killable.

Let $p \in {\mathcal {T}} ({\mathcal {S}})$ be a $\pi$-witness to ${\mathcal {S}} ^{c(m)} \models \phi _3({\mathcal {I}},{\mathcal {O}})$. First, we show that traces of ${\mathcal {S}} ^m$ with inputs $p\vert _{{{\mathcal {I}}}}$ can not repeat before having a different output to *p*. Assume the contrary, i.e., there are $q \in {\mathcal {T}} ({\mathcal {S}} ^m)$ and $l < j \le k$, such that $q\vert _{{{\mathcal {I}}}} = p\vert _{{{\mathcal {I}}}}, q[0,k]\vert _{{{\mathcal {O}}}} = p[0,k]\vert _{{{\mathcal {O}}}},$ and $q[l] = q[j]$. Trace *q* can be modified to a trace that loops between *q*[*l*] and *q*[*j*] indefinitely. This trace is a counter-example to ${\mathcal {S}} ^m$ being definitely killable. Let *n* be the finite number of different non-repeating prefixes of traces of ${\mathcal {S}} ^m$. Clearly, the test $t {\mathop {=}\limits ^{\tiny def }}p[0,n]\vert _{{{\mathcal {I}} \cup {\mathcal {O}}}}$ definitely kills ${\mathcal {S}} ^m$. $\square$

In the case of infinite systems, there might be a definitely killable mutant for which no (finite) definitely killing test exists.

#### Example 4

Consider the following infinite version of the beverage machine, where capacity=* denotes non-deterministically choosing some integer value capacity $\in {\mathbb {N}}$. The volume of the water tank is fixed to this value. Otherwise, the system behaves similarly to the system with a definitely killable mutant presented in Example 1. In particular, clearly the presented mutant is definitely killable, since after filling the water tank, the mutant will not produce a beverage upon capacity more beverage requests. However, since capacity is chosen non-deterministically by the system, there is no universal length for a finite test that would reveal this behavior. The infinite trace that continuously requests beverages after filling could be considered an infinite killing test though.

$$\begin{aligned} \begin{aligned} \alpha {\mathop {=}\limits ^{\tiny def }}&{{{\mathbf {\mathtt{{capacity=*}}}}}} \wedge \texttt {out=}\varepsilon \wedge {{{\mathbf {\mathtt{{wtr=capacity}}}}}} \\ \delta {\mathop {=}\limits ^{\tiny def }}&{{{\mathbf {\mathtt{{capacity'=capacity}}}}}} \wedge \\&(\texttt {in=req} \wedge \texttt {wtr>0} \wedge \texttt {out=coff} \wedge \texttt {wtr'=wtr-1} \vee \\&\texttt {in=req} \wedge \texttt {wtr>0} \wedge \texttt {out=tea} \wedge \texttt {wtr'=wtr-1} \vee \\&\texttt {in=fill} \wedge \texttt {wtr=0} \wedge \lnot \texttt {mut} \wedge \texttt {out=}\varepsilon \wedge {{{\mathbf {\mathtt{{wtr'=capacity}}}}}} \vee \\&\texttt {in=fill} \wedge \texttt {wtr=0} \wedge \texttt {mut} \wedge \texttt {out=}\varepsilon \wedge {{{\mathbf {\mathtt{{wtr'=capacity-1}}}}}} \vee \\&\texttt {in=}\varepsilon \wedge \texttt {out=}\varepsilon \wedge \texttt {wtr'=wtr}) \end{aligned} \end{aligned}$$

### Mixed determinism model and mutant

We now examine cases where the model is non-deterministic and the mutant is deterministic and vice versa. It should be noted that in practice it might not be known a priori whether a model or mutant is really deterministic. In such cases, the hyperproperties $\phi _2({\mathcal {I}},{\mathcal {O}})$ and $\phi _3({\mathcal {I}},{\mathcal {O}})$ for non-deterministic mutants can be used to define and construct killing test cases, as their guarantees hold for deterministic mutants as well. Nevertheless, in this section, we present the weakest hyperproperties expressing potential and definite killability for mixed determinism cases.

To show potential killability of a non-deterministic mutant for a deterministic model, one needs to find a trace of the model ($\exists \pi$) such that there is a trace of the mutant with the same inputs ($\exists \pi '$) that eventually diverges in outputs, which is exactly formalized by the hyperproperty $\phi _1$ above.

#### Proposition 5

Let the model ${\mathcal {S}}$ with inputs ${\mathcal {I}}$ and outputs ${\mathcal {O}}$ be deterministic and the mutant ${\mathcal {S}} ^m$ be non-deterministic.

$$\begin{aligned} {\mathcal {S}} ^{c(m)} \models \phi _1({\mathcal {I}},{\mathcal {O}})\text { iff } {\mathcal {S}} ^m \text { is potentially killable.} \end{aligned}$$

Let *p* be a $\pi$-witness for ${\mathcal {S}} ^{c(m)} \models \phi _1({\mathcal {I}},{\mathcal {O}})$, then there exists $n \in {\mathbb {N}}$ such that test $t {\mathop {=}\limits ^{\tiny def }}p[0,n]\vert _{{{\mathcal {I}} \cup {\mathcal {O}}}}$ potentially kills ${\mathcal {S}} ^m$.

#### Proof

The proof can be conducted similar to the proof of Proposition 2. $\square$

To show definite killing of a non-determistic mutant of a deterministic model, one needs to find a trace of the model ($\exists \pi$) such that all traces of the mutant with the same inputs ($\forall \pi '$) eventually diverge in outputs, which is formalized via the hyperproperty $\phi _4$ as follows:

$$\begin{aligned} \phi _4({\mathcal {I}},{\mathcal {O}}) {\mathop {=}\limits ^{\tiny def }}\exists \pi \forall \pi ' \Box \lnot \mathrm {mut}_{\pi } \wedge \Big (\Box \big (\mathrm {mut}_{\pi '} \wedge (I_{\pi } \leftrightarrow I_{\pi '})\big ) \rightarrow \lozenge \big (O_{\pi } \not \leftrightarrow O_{\pi '}\big )\Big ) \end{aligned}$$

#### Proposition 6

Let the model ${\mathcal {S}}$ with inputs ${\mathcal {I}}$ and outputs ${\mathcal {O}}$ be deterministic and the mutant ${\mathcal {S}} ^m$ be non-deterministic.

$$\begin{aligned} {\mathcal {S}} ^{c(m)} \models \phi _4({\mathcal {I}},{\mathcal {O}})\text { iff } {\mathcal {S}} ^m \text { is definitely killable.} \end{aligned}$$

If ${\mathcal {S}} ^m$ is finite and *p* is a $\pi$-witness for ${\mathcal {S}} ^{c(m)} \models \phi _4({\mathcal {I}},{\mathcal {O}})$, then there exists $n \in {\mathbb {N}}$ such that the test $t {\mathop {=}\limits ^{\tiny def }}p[0,n]\vert _{{{\mathcal {I}} \cup {\mathcal {O}}}}$ definitely kills ${\mathcal {S}} ^m$.

#### Proof

Assume that ${\mathcal {S}} ^m$ is definitely killable. Since ${\mathcal {S}}$ is deterministic, for every input sequence, there is at most one trace with in ${\mathcal {T}} ({\mathcal {S}})$ with this input sequence. Therefore, there is an input sequence $\vec {I}$ and a unique trace $p \in {\mathcal {T}} ({\mathcal {S}})$ with $p_I = \vec {I}$ and $p\vert _{{{\mathcal {I}} \cup {\mathcal {O}}}} \notin {\mathcal {T}} ({\mathcal {S}} ^m)\vert _{{{\mathcal {I}} \cup {\mathcal {O}}}}$. Any trace assignment that maps $\pi$ to *p* satisfies $\phi _4({\mathcal {I}},{\mathcal {O}})$, since either the antecedent is violated by a trace $q \in {\mathcal {T}} ({\mathcal {S}} ^m)$ assigned to $\pi '$ with different inputs, or the consequent is violated by a trace $q \in {\mathcal {T}} ({\mathcal {S}} ^m)$ assigned to $\pi '$ with inputs $\vec {I}$ and outputs that can only be different to $p\vert _{{{\mathcal {O}}}}$.

Conversely, assume ${\mathcal {S}} ^{c(m)} \models \phi _4({\mathcal {I}},{\mathcal {O}})$. Let $\varPi$ be a satisfying trace assignment that maps $\pi$ to *q* and $\pi '$ to *p* that also satisfies the antecedent, which is in fact satisfiable, since ${\mathcal {S}} ^m$ is equally input-enabled. Clearly, *p* is a $\pi$-witness for $\Box \lnot \mathrm {mut}_{\pi }$ and since *q* was chosen such that it satisfies the antecedent, *q* is a $\pi '$-witness for $\Box \mathrm {mut}_{\pi '}$. Thus, from Lemma 1, we get $p\vert _{{{\mathcal {I}} \cup {\mathcal {O}} \cup {\mathcal {X}}}} \in {\mathcal {T}} ({\mathcal {S}})$, $q\vert _{{{\mathcal {I}} \cup {\mathcal {O}} \cup {\mathcal {X}}}} \in {\mathcal {T}} ({\mathcal {S}} ^m)$, and $q\vert _{{{\mathcal {I}}}} = p\vert _{{{\mathcal {I}}}}$. Since $\varPi$ satisfies the whole formula, it must be the case that $\varPi$ also satisfies the consequent, i.e. $q\vert _{{{\mathcal {O}}}} \ne p\vert _{{{\mathcal {O}}}}$ (Lemma 1). Therefore, we can conclude $p\vert _{{{\mathcal {I}} \cup {\mathcal {O}}}} \notin {\mathcal {T}} ({\mathcal {S}} ^m)\vert _{{{\mathcal {I}} \cup {\mathcal {O}}}}$, which, as argued above, is equivalent to definite killing in the deterministic model case.

The existence of a definitely killing test in case of finite ${\mathcal {S}} ^m$ can be shown analogously to the proof of Proposition 4. $\square$

Finally, to show killability of a deterministic mutant for a non-deterministic model, one needs to find a trace of the mutant ($\exists \pi$) such that all traces of the model with the same inputs ($\forall \pi '$) eventually diverge in outputs, which is already expressed via the hyperproperty $\phi _2$ above.

#### Proposition 7

Let the model ${\mathcal {S}}$ with inputs ${\mathcal {I}}$ and outputs ${\mathcal {O}}$ be non-deterministic and the mutant ${\mathcal {S}} ^m$ be deterministic

$$\begin{aligned} {\mathcal {S}} ^{c(m)} \models \phi _2({\mathcal {I}},{\mathcal {O}}) \text { iff } {\mathcal {S}} ^m \text { is killable}. \end{aligned}$$

Let *q* be a $\pi$-witness for ${\mathcal {S}} ^{c(m)} \models \phi _2({\mathcal {I}},{\mathcal {O}})$, then there is $n \in {\mathbb {N}}$, such for the single trace $p \in {\mathcal {T}} ({\mathcal {S}})$ with $p\vert _{{{\mathcal {I}}}} = q\vert _{{{\mathcal {I}}}}$ the test $t {:}{=} p[0,n]\vert _{{{\mathcal {I}} \cup {\mathcal {O}}}}$ kills ${\mathcal {S}} ^m$.

#### Proof

Potential killing directly follows from the more restricted case in Proposition 3. Since ${\mathcal {S}} ^m$ is deterministic, by Proposition 1 it is also definitely killable. The existence of a killing test can be shown analogously to the proof of Proposition 3. $\square$

### Locally adaptive tests

We can extend the hyperproperties presented above to force $\pi$-witnesses to have prefixes that are locally adaptive tests. To this end, we need to reason over the allowed output indicator variables in ${\mathcal {A}}$. So far, these variables are unconstrained. However, we can strengthen the hyperproperties expressing killability, such that only assignments to these variables are allowed that reflect the semantics of allowed outputs. Unfortunately, these semantics are not expressible in HyperLTL, since they require to reason over all outgoing traces from intermediate states of an arbitrary trace. However, the property is expressible in HyperCTL*.

For an STS with a finite set of outputs $\mathop {Out}$ and a HyperLTL formula $\phi$ of the form $\exists \pi \psi$, we define its locally adaptive test extension $\phi ^{{\mathcal {A}}}$ as:

$$\begin{aligned} \phi ^{{\mathcal {A}}} {\mathop {=}\limits ^{\tiny def }}\exists \pi \bigg (\psi \wedge \bigwedge _{O \in \mathop {Out}} \Box \mathrm {X}\Big (a[O]_{\pi } \leftrightarrow&\big (\exists \pi ' \lnot \mathrm {mut}_{\pi '} \wedge (I_{\pi } \leftrightarrow I_{\pi '}) \wedge \\&\bigwedge _{o \in \mathsf {AP} (O)} o_{\pi '} \wedge \bigwedge _{o \in \mathsf {AP} ({\mathcal {O}}) \setminus \mathsf {AP} (O)} \lnot o_{\pi '}\big )\Big )\bigg ) \end{aligned}$$

For example, for $\phi _3({\mathcal {I}},{\mathcal {O}})$ the full extended formula is given as follows:

$$\begin{aligned}&\phi _3({\mathcal {I}},{\mathcal {O}})^{{\mathcal {A}}} {\mathop {=}\limits ^{\tiny def }}\\&\quad \exists \pi \bigg (\forall \pi ' \forall \pi '' \Box \lnot \mathrm {mut}_{\pi }\\&\quad \wedge \Big (\Box \big (\mathrm {mut}_{\pi '} \wedge \lnot \mathrm {mut}_{\pi ''} \wedge (I_{\pi } \leftrightarrow I_{\pi '}) \wedge (I_{\pi } \leftrightarrow I_{\pi ''})\big ) \rightarrow \\&\quad \lozenge \big (O_{\pi '} \not \leftrightarrow O_{\pi ''}\big )\Big ) \wedge \\&\quad \bigwedge _{O \in \mathop {Out}} \Box \mathrm {X}\Big (a[O]_{\pi } \leftrightarrow \big (\exists \pi ' \lnot \mathrm {mut}_{\pi '} \wedge (I_{\pi } \leftrightarrow I_{\pi '}) \wedge \\&\quad \bigwedge _{o \in \mathsf {AP} (O)} o_{\pi '} \wedge \bigwedge _{o \in \mathsf {AP} ({\mathcal {O}}) \setminus \mathsf {AP} (O)} \lnot o_{\pi '}\big )\Big )\bigg ) \end{aligned}$$

Likewise, this extension can be performed for $\phi _1({\mathcal {I}},{\mathcal {O}})$ and $\phi _4({\mathcal {I}},{\mathcal {O}})$. Note that the $\pi$ path variable in $\phi _2({\mathcal {I}},{\mathcal {O}})$ is constrained to evaluate to paths of the mutant. Thus, in order to leverage this transformation for $\phi _2({\mathcal {I}},{\mathcal {O}})$, an additional existential quantifier picking one suitable trace of the original STS needs to be added to the formula.

We now show that models of these extensions contain locally adaptive tests.

#### Proposition 8

Let ${\mathcal {S}} ^{c(m)}$ be a conditional mutant and let $\phi$ be a HyperLTL formula of the form $\exists \pi \psi$ such that ${\mathcal {S}} ^{c(m)} \models \psi$ and some finite prefix of a $\pi$-witness to ${\mathcal {S}} ^{c(m)} \models \psi$ is a linear test, then ${\mathcal {S}} ^{c(m)} \models ^* \phi ^{{\mathcal {A}}}$ and some finite prefix of the trace component of a $\pi ^*$-witness for ${\mathcal {S}} ^{c(m)} \models ^* \phi ^{{\mathcal {A}}}$ is a locally adaptive test.

#### Proof

Let $p \in {\mathcal {T}} ({\mathcal {S}})$ be a $\pi$-witness to ${\mathcal {S}} ^{c(m)} \models \psi$ and let $t {\mathop {=}\limits ^{\tiny def }}p[0,n]$ be the finite prefix that is a linear test. Since variables *a*[*O*] are unconstrained by the STS, we can assume that the valuations of these variables in *p* are chosen such that *p* (together with its states) constitutes a $\pi ^*$-witness for ${\mathcal {S}} ^{c(m)} \models ^* \phi ^{{\mathcal {A}}}$. By assumption, there is only one initial state only one unique output in the STS, so there is nothing to show in the initial step. To show that *t* is a locally adaptive test, it needs to be the case that for every $j \in [1,n]$ and every $O \in \mathop {Out}$ it is the case that *t*[*j*] at *a*[*O*] evaluates to $\top$ if and only if there exists a trace $p' \in {\mathcal {T}} ({\mathcal {S}})$ with $p'[0,i-1]~=~t[0,i-1]$, $p'[j]\vert _{{{\mathcal {I}}}}~=~t[j]\vert _{{{\mathcal {I}}}}$, and $p'[j]\vert _{{{\mathcal {O}}}}~=~O$. Path *p* is chosen such that in every step $j-1$ and output *O*, *p* evaluates *a*[*O*] to $\top$ in its successor state if and only if from the current state there a path of the original system $p^O_j$ whose next state exactly has input $p[j]\vert _{{{\mathcal {I}}}}$ and output *O*. Therefore, paths $p^O_j$ for every $O \in \mathop {Out}$, prepended with the prefix of *p* up to *j*, are witnesses to this property. $\square$

Unfortunately, to the best of the knowledge of the authors, there currently does not exists a model checker for HyperCTL*. However, the problem was shown to be decidable in [31], although its complexity grows exponentially in the number of quantifier alternations. Therefore, on top of providing formal semantics for locally adaptive tests, the encoding can be leveraged in practice soon as a HyperCTL* model checker emerges.

## Non-deterministic models in practice

Checking the validity of the hyperproperties in Sect. 4 for a given model and mutant enables test-case generation. Unfortunately, the model checkers for HyperLTL or HyperCTL* are still in their infancy. To the best of our knowledge, MCHyper [31] is the only currently available HyperLTL model checker and there is no HyperCTL* model checker. Furthermore, HyperLTL formulas with quantifier alternation, such as killability defining formulas $\phi _2({\mathcal {I}},{\mathcal {O}})$ and $\phi _3({\mathcal {I}},{\mathcal {O}})$ for non-deterministic models, can currently not be handled with the available version of the tool. In a Web-based version of MCHyper such formulas can be handled via a combination with a reactive synthesis tool, as described in [21]. To remedy this issue and to obtain test cases for non-deterministic systems, in this section, we propose two solutions.

Firstly, we present a transformation that makes non-determinism *controllable* by means of additional inputs and yields a deterministic STS. The quantifier alternation free formula $\phi _1({\mathcal {I}},{\mathcal {O}})$ can be model-checked over the transformed model. The result is an over-approximation of killability in the sense that the resulting test cases only kill some non-deterministic mutant if non-determinism can also be controlled in the system under test. However, if equivalence can be established for the transformed model, then the non-deterministic mutant is also equivalent. In Sect. 5.1 we define the transformation formally and prove its properties. In Sect. 5.2, we show how the transformation can be done syntactically in practice.

Secondly, we propose an encoding of model-checking $\phi _2({\mathcal {I}},{\mathcal {O}})$ and $\phi _3({\mathcal {I}},{\mathcal {O}})$ into a bounded SMT satisfiability problem. This problem can be solved with off-the-shelf solvers such as the SMT solver Z3 [22] or the first-order logic solver Vampire [42].

### Controlling non-determinism in STS

The essential idea of our transformation is to introduce an additional *input* (represented by an auxiliary variable *nd*) that enables the control of non-deterministic choices in a conditional mutant ${\mathcal {S}} ^{c(m)}$ with finite non-deterministic branching. The new input is used carefully to ensure that choices are consistent for the model and the mutant encoded in ${\mathcal {S}} ^{c(m)}$. Without loss of generality, we assume that variable *nd* has a finite range sufficiently large to encode the non-deterministic choices in $\alpha ^{c(m)}$ and $\delta ^{c(m)}$. We use abbreviations $nd(X,I,O,X')$, $nd(X,O,X')$ for $nd = n_{X,I,O,X'}$, $nd = n_{X,O,X'}$ and values $n_{X,I,O,X'}$, $n_{X,O,X'}$ in the range of *nd* that uniquely correspond to the non-deterministic choice of output *O* and successor state $X'$ from state *X* and in response to input *I*, any input, respectively. Moreover, we add a fresh Boolean variable $x^{\tau }$ to ${\mathcal {X}}$ that we use to encode a fresh initial state.

Let ${\mathcal {X}} _+ {\mathop {=}\limits ^{\tiny def }}{\mathcal {X}} \cup \{\mathrm {mut}\}$ and $X_{+},X'_{+},I,O$ be valuations of ${\mathcal {X}} _{+}$, ${\mathcal {X}} '_{+}$, ${\mathcal {I}}$, and ${\mathcal {O}}$, and *X* and $X'$ denote ${X_+}\vert _{{{\mathcal {X}}}}$ and ${X_+'}\vert _{{{\mathcal {X}} '}}$, respectively. Furthermore, $\psi (X)$, $\psi (X_+,I)$, and $\psi (O,X'_+)$ are formulas uniquely satisfied by *X*, $(X_+,I)$, and $(O,X'_+)$, respectively.

Given conditional mutant ${\mathcal {S}} ^{c(m)} {\mathop {=}\limits ^{\tiny def }}\langle {\mathcal {I}},{\mathcal {O}},{\mathcal {X}} _+,\alpha ^{c(m)},\delta ^{c(m)} \rangle$, we define its controllable counterpart $D({\mathcal {S}} ^{c(m)}) {\mathop {=}\limits ^{\tiny def }}\langle {\mathcal {I}} \cup \{nd\},{\mathcal {O}},{\mathcal {X}} _+ \cup \{x^{\tau }\},D(\alpha ^{c(m)}),D(\delta ^{c(m)}) \rangle$. We initialize $D(\delta ^{c(m)}) {\mathop {=}\limits ^{\tiny def }}\delta ^{c(m)}$ and incrementally add constraints as described below.

*Non-deterministic initial conditions:* Let *X* be an arbitrary, fixed state. The unique fresh initial state is $X^{\tau } {\mathop {=}\limits ^{\tiny def }}X[x^{\tau } \mapsto \top ]$, which, together with an empty output, we enforce by the new initial conditions predicate:

$$\begin{aligned} D(\alpha ^{c(m)}) {\mathop {=}\limits ^{\tiny def }}\psi (X^{\tau }, O_{\varepsilon }) \end{aligned}$$

We add the conjunct $\lnot \psi (X^{\tau }) \rightarrow \lnot {x^{\tau }}'$ to $D(\delta ^{c(m)})$, in order to force $x^{\tau }$ evaluating to $\bot$ in all states other than $X^{\tau }$. In addition, we add transitions from $X^{\tau }$ to all pairs of initial states/outputs in $\alpha ^{c(m)}$. To this end, we first partition the pairs in $\alpha ^{c(m)}$ into pairs shared by and exclusive to the model and the mutant:

$$\begin{aligned} \begin{array}{lll} J^{\cap } &{}{\mathop {=}\limits ^{\tiny def }}\{(O,X_+) \mid &{}X,O \models \alpha ^{c(m)}\}\\ J^{orig} &{}{\mathop {=}\limits ^{\tiny def }}\{(O,X_+) \mid &{}\lnot X_+(\mathrm {mut}) \wedge (X_+,O \models \alpha ^{c(m)}) \wedge \\ &{} &{}(X_+[\mathrm {mut} \mapsto \top ],O \not \models \alpha ^{c(m)})\}\\ J^{mut} &{}{\mathop {=}\limits ^{\tiny def }}\{(O,X_+) \mid &{}X_+(\mathrm {mut}) \wedge (X_+,O \models \alpha ^{c(m)}) \wedge \\ &{} &{}(X_+[\mathrm {mut} \mapsto \bot ],O \not \models \alpha ^{c(m)})\}\\ \end{array} \end{aligned}$$

For each $(O,X_+) \in J^{\cap } \cup J^{mut} \cup J^{orig}$, we add the following conjunct to $D(\delta ^{c(m)})$:

$$\begin{aligned} \psi (X^{\tau }) \wedge nd(X^{\tau },O,X') \rightarrow \psi (O,X_+') \end{aligned}$$

In addition, in order to retain that the model and mutant are equally input-enabled, for outputs *O* and successor states $X'$ without corresponding non-deterministic choice in the model or mutant, we add conjuncts to $D(\delta ^{c(m)})$ that represent transitions with empty outputs to the respective successor state:

$$\begin{aligned} \begin{array}{ll} \forall (O,X_+) \in J^{orig}: &{}\left( \psi (X^{\tau }[\mathrm {mut} \mapsto \top ]]) \wedge nd(X^{\tau },O,X')\right) \rightarrow \\ &{}\psi (O_{\varepsilon },{X^{\tau }}'[\mathrm {mut} \mapsto \top ]) \\ \forall (O,X_+) \in J^{mut}: &{}\left( \psi (X^{\tau }[\mathrm {mut} \mapsto \bot ]) \wedge nd(X^{\tau },O,X')\right) \rightarrow \\ &{}\psi (O_{\varepsilon },{X^{\tau }}'[\mathrm {mut} \mapsto \bot ]) \end{array} \end{aligned}$$

*Non-deterministic transitions:* Analogous to initial states, for each state/input pair, we partition the successors into successors shared or exclusive to model or mutant:

$$\begin{aligned} \begin{array}{lll} T^{\cap }_{(X_+,I)} &{} {\mathop {=}\limits ^{\tiny def }}\{&{}(X_+,I,O,X_+') \mid X \xrightarrow {I,O} X'\} \\ T^{orig}_{(X_+,I)} &{} {\mathop {=}\limits ^{\tiny def }}\{&{}(X_+,I,O,X_+') \mid \\ &{} &{} \lnot X_+(\mathrm {mut}) \wedge (X_+ \xrightarrow {I,O} X_+') \wedge \lnot (X_+[\mathrm {mut} \mapsto \top ] \xrightarrow {I,O} X_+')\} \\ T^{mut}_{(X_+,I)} &{} {\mathop {=}\limits ^{\tiny def }}\{&{}(X_+,I,O,X_+') \mid \\ &{} &{}X_+(\mathrm {mut}) \wedge (X_+ \xrightarrow {I,O} X_+') \wedge \lnot (X_+[\mathrm {mut} \mapsto \bot ] \xrightarrow {I,O} X_+')\} \\ \end{array} \end{aligned}$$

A pair $(X_+,I)$ causes non-determinism if

$$\begin{aligned}&|(T^{\cap }_{(X_+,I)}~\cup ~T^{orig}_{(X_+,I)})\vert _{{{\mathcal {X}} \cup {\mathcal {I}} \cup {\mathcal {O}} \cup {\mathcal {X}} '}}|\\&> 1 \text { or } |(T^{\cap }_{(X_+,I)}~\cup ~T^{mut}_{(X_+,I)})\vert _{{{\mathcal {X}} \cup {\mathcal {I}} \cup {\mathcal {O}} \cup {\mathcal {X}} '}}|>1. \end{aligned}$$

For each pair $(X_+,I)$ that causes non-determinism and each $(X_+,I,O_j,{X_{+j}'}) \in T^{\cap }_{(X_+,I)} \cup T^{mut}_{(X_+,I)} \cup T^{orig}_{(X_+,I)}$, we add the following conjunct to $D(\delta ^{c(m)})$:

$$\begin{aligned} \psi (X_+,I) \wedge nd(X,I,O_j,X'_j) \rightarrow \psi (O_j,{X_{+j}'}) \end{aligned}$$

Finally, to retain that model and mutant are equally input-enabled, we add conjuncts representing transitions with empty output for non-deterministic choices that have no corresponding transition in the model or mutant:

$$\begin{aligned}&\forall (X_+,I,O_j,{X_{+j}'}) \in T^{orig}_{(X_+,I)}:\\&\left( \psi (X_+[\mathrm {mut} \mapsto \bot ],I) \wedge nd(X,I,O_j,{X'_j})\right) \rightarrow \\&\psi (O_{\varepsilon },X_{+j}'[\mathrm {mut} \mapsto \top ]) \\&\forall (X_+,I,O_j,{X_{+j}'}) \in T^{mut}_{(X_+,I)}:\\&\left( \psi (X_+[\mathrm {mut} \mapsto \bot ],I) \wedge nd(X,I,O_j,{X'_j})\right) \rightarrow \\&\psi (O_{\varepsilon },X_{+j}'[\mathrm {mut} \mapsto \bot ]) \end{aligned}$$

The proposed transformation has the following properties:

#### Proposition 9

Let ${\mathcal {S}}$ be a model with inputs ${\mathcal {I}}$, outputs ${\mathcal {O}}$, mutant ${\mathcal {S}} ^m$, and finite non-deterministic branching then

1. $D({\mathcal {S}} ^{c(m)})$ is deterministic (up to $\mathrm {mut}$).
2. $${\mathcal {T}} ({\mathcal {S}} ^{c(m)})\vert _{{{\mathcal {X}} _+ \cup {\mathcal {I}} \cup {\mathcal {O}}}} \subseteq {\mathcal {T}} (D({\mathcal {S}} ^{c(m)}))[1,\infty ]\vert _{{{\mathcal {X}} _+ \cup {\mathcal {I}} \cup {\mathcal {O}}}}$$.
3. $D({\mathcal {S}} ^{c(m)}) \not \models \phi _1({\mathcal {I}},{\mathcal {O}})$ then ${\mathcal {S}} ^m$ is equivalent.

#### Proof

Statement 1: We show $D({\mathcal {S}} ^{c(m)})$ is deterministic (up to $\mathrm {mut}$). $D({\mathcal {S}} ^{c(m)})$ has a unique (up to $\mathrm {mut}$) initial state $X^{\tau }$ and initial output $O_{\varepsilon }$, since we fix $D(\alpha ^{c(m)})$ to be satisfiable by exactly this state and output.

Next we show that an state/input pair (*X*, *I*) uniquely fixes output *O* and successor state $X'$ in $D({\mathcal {S}} ^{c(m)})$. Firstly, due to the conjunct $\lnot \psi (X^{\tau }) \rightarrow \lnot {x^{\tau }}'$, the value of $x^{\tau }$ is fixed throughout the transition system. Secondly, consider the case where (*X*, *I*) does not cause non-determinism in ${\mathcal {S}}$. Then, no constraint for state *X* and input *I* is introduced to $D(\delta ^{c(m)})$, i.e. every value for *nd* either leads to the same successor output and state or not such transition is possible at all. Thirdly, consider the case where (*X*, *I*) causes non-determinism in ${\mathcal {S}}$. For such pairs, we enumerate all finitely many possible successor outputs and states and conjunctively add implications with antecedents that are satisfied by unique values of values of *nd*. Therefore, only one conclusion of the form $\psi (O_j,{X_{+j}'})$ can be satisfied by a given pair of state *X* and input *I*.

Statement 2: We show $p \in {\mathcal {T}} ({\mathcal {S}} ^{c(m)})\vert _{{{\mathcal {X}} _+ \cup {\mathcal {I}} \cup {\mathcal {O}}}}[0,n]$ then $p \in {\mathcal {T}} (D({\mathcal {S}} ^{c(m)}))[1,n+1]\vert _{{{\mathcal {X}} _+ \cup {\mathcal {I}} \cup {\mathcal {O}}}}$ by induction on *n*.

First note that sets $J^{\cap }$, $J^{orig}$, and $J^{mut}$ are pairwise disjoint and contain every initial state/output pair of ${\mathcal {S}} ^{c(m)}$, since we use the very definition of initial state/output pairs to define those sets, possibly splitting them according to values of *mut*. Likewise, for each (*X*, *I*) sets $T^{\cap }_{(X,I)} \cup T^{mut}_{(X,I)} \cup T^{orig}_{(X,I)}$ are pairwise disjoint and contain every transition for (*X*, *I*) as a tuple $(X,I,O,X')$.

In the base case, $n = 0$, let $p = (I_0,X_0,O_0)$, where $X_0$ and $O_0$ are initial state and output of ${\mathcal {S}} ^{c(m)}$. We need to show that there is a trace $q \in {\mathcal {T}} (D({\mathcal {S}} ^{c(m)}))$, such that $q[1]\vert _{{{\mathcal {X}} _m \cup {\mathcal {I}} \cup {\mathcal {O}}}} = (I_0,X_0,O_0)$. As noted above $(O_0,X_0) \in J^{\cap } \cup J^{orig} \cup J^{mut}$. Therefore, we add a constraint corresponding to a transition $X^{\tau } \xrightarrow {nd(X^{\tau },O_0,X_0),O_0} X'_0$ to the system. Furthermore, for $(X_0,I_0)$, we add a constraint corresponding to a transition $X_0 \xrightarrow {I_0,O} X'$ for some output *O* and successor $X'$. Therefore, the trace *q* exists.

In the inductive step, assume that the statement holds for $n-1$ and consider the case for *n*. Let $p[n-1] = (I_{n-1},X_{n-1},O_{n-1})$ and $p[n] = (I_{n},X_{n},O_{n})$. We need to show that for some trace $q \in {\mathcal {T}} (D({\mathcal {S}} ^{c(m)}))$ with $q[n]\vert _{{{\mathcal {X}} _m \cup {\mathcal {I}} \cup {\mathcal {O}}}} = (I_{n-1},X_{n-1},O_{n-1})$—which exists due to the induction hypothesis—it is the case that $q\vert _{{{\mathcal {X}} _m \cup {\mathcal {I}} \cup {\mathcal {O}}}}[n+1] = (I_{n},X_{n},O_{n})$. Since $p \in {\mathcal {T}} ({\mathcal {S}} ^{c(m)})$ it is the case that $X_{n-1} \xrightarrow {I_{n-1},O_n} X_{n}'$ in ${\mathcal {S}} ^{c(m)}$. In case $(X_{n-1},I_{n-1})$ does not cause non-determinism, no constraints are added to the transition system and $X_{n-1} \xrightarrow {I_{n-1},O_n} X_{n}'$ in $D({\mathcal {S}})^{c(m)}$. In case $(X_{n-1},I_{n-1})$ causes non-determinism, transitions are exhaustively enumerated via distinction on values of the fresh *nd* variable, i.e., $X_{n-1} \xrightarrow {I_{n-1}\cup \{nd(X_{n-1},I_{n-1},O_n,X'_n)\},O_n} X_{n}'$ in $D({\mathcal {S}})^{c(m)}$.

Note also that a consequence of the above statements, and the fact that we introduce transitions for different values of *nd* exhaustively, is that $D({\mathcal {S}} ^{c(m)})$ preserves equal input-enabledness.

Statement 3: $\not \models \phi _1({\mathcal {I}},{\mathcal {O}})$ then ${\mathcal {S}} ^m$ is equivalent is a direct consequence of the statements about traces, since $\not \models \phi _1({\mathcal {I}},{\mathcal {O}})$ shows no trace in ${\mathcal {T}} (D({\mathcal {S}} ^{c(m)}))$ is a witness to killing the mutant. Since traces of ${\mathcal {S}} ^{c(m)}$ are included in (the projection of) this set, there can not be a trace in ${\mathcal {T}} ({\mathcal {S}} ^{c(m)})$ that is a witness to killing the mutant. $\square$

In summary, the transformed model is deterministic, since we enforce unique initial valuations and make non-deterministic transitions controllable through input *nd*. Since we only add transitions or augment existing transitions with input *nd*, every transition $X \xrightarrow {I,O} X'$ of ${\mathcal {S}} ^{c(m)}$ is still present in $D({\mathcal {S}} ^{c(m)})$ (when input *nd* is disregarded). The potential additional traces of Statement 2 originate from the $O_{\varepsilon }$-labeled transitions for non-deterministic choices present exclusively in the model or mutant. These transitions enable the detection of discrepancies between model and mutant caused by the introduction or elimination of non-determinism by the mutation.

Statement 3 shows what can be achieved by model checking the quantifier alternation free formula $\phi _1$ over the transformed controllable determinism STS $D({\mathcal {S}} ^{c(m)})$. Equivalent mutants of this system are also equivalent in the non-deterministic version. Killability purported by $\phi _1$, however, could be an artifact of the transformation: Determinization potentially deprives the model of its ability to match the output of the mutant by deliberately choosing a certain non-deterministic transition. Test cases can therefore only be considered killing under the assumption that non-determinism can be controlled by the tester. In Example 3, we present an equivalent mutant which is killable after the transformation, since we will detect the deviating output tea of the model and $\varepsilon$ of the mutant. Therefore, our transformation merely allows us to provide a lower bound for the number of equivalent non-deterministic mutants.

### Controlling non-determinism in modeling languages

The exhaustive enumeration of states (*J*) and transitions (*T*) outlined in Sect. 5.1 is purely theoretical and infeasible in practice. However, an analogous result can often be achieved by modifying the syntactic constructs of the underlying modeling language that introduce non-determinism, namely:

- *Non-deterministic assignments.* Non-deterministic choice over a finite set of elements $\{x'_1,\ldots x'_n\}$, as provided by SMV [46], can readily be converted into a case-switch construct over *nd*. More generally, explicit non-deterministic assignments $\mathtt{x {:}{=} \star }$ to state variables x [48] can be controlled by assigning the value of *nd* to x.
- *Non-deterministic schedulers.* Non-determinism introduced by concurrency can be controlled by introducing input variables that control the scheduler (as proposed in [43] for bounded context switches).

In case non-determinism arises through variables under-specified in transition relations, these variable values can be made inputs as suggested by Sect. 5.1. In general, however, identifying under-specified variables automatically is non-trivial.

#### Example 5

Consider again the SMV code in Fig. 1a, for which non-determinism can be made controllable by replacing **if**(in=req&wtr>0):{coff,tea} with **if**(nd=0&in=req&wtr>0):coff **elif**(nd=1&in=req&wtr>0):tea and adding **init**(nd):={0,1}.

Similarly, the STS representation of the beverage machine, given in Example 1, can be transformed by replacing the first two rules by the following two rules:

$$\begin{aligned}&\texttt {nd=0} \wedge \texttt {wtr>0} \wedge \texttt {in=req} \wedge \texttt {out=coff} \wedge \texttt {wtr'=wtr-1} \vee \\&\texttt {nd=1} \wedge \texttt {wtr>0} \wedge \texttt {in=req} \wedge \texttt {out=tea} \wedge \texttt {wtr'=wtr-1} \vee \end{aligned}$$

Finally, the results of the transformation is presented in Fig. 6 on a case study.

### Encoding bounded killability into SMT

Another way of solving killability properties with quantifier alternation is to leverage the first order expressability of HyperLTL (proven in [32]) and to encode the problem into a suitable fragment of first order logic. Let ${\mathcal {S}} ^{c(m)} {\mathop {=}\limits ^{\tiny def }}\langle {\mathcal {I}},{\mathcal {O}},{\mathcal {X}} _+,\alpha ^{c(m)},\delta ^{c(m)} \rangle$ be a conditional mutant, where for ease of presentation, we abbreviate $\alpha ^{c(m)}$ with $\alpha$ and $\delta ^{c(m)}$ with $\delta$ throughout this section. We describe an SMT encoding of potential and definite killability into a bounded (up to fixed bound *k*) satisfiability problem in a logic that contains the logic of the symbolic transition system, as well as quantification over the ranges of state and output variables. The idea is to create copies of each variable and each step for the mutant as well as the original. Furthermore, the transition relation is replicated for each step, using the respective step variables. Reachable states and outputs are expressed by universally quantifying over variable values and checking whether the respective variable assignment satisfies the initial state-, as well as, the step-wise transition relation- predicate.

Let ${\mathcal {I}} = \{i_0,\ldots ,i_{m_i}\}$, ${\mathcal {O}} = \{o_0,\ldots ,o_{m_o}\}$, and ${\mathcal {X}} = \{x_0,\ldots ,x_{m_x}\}$. For each variable $i \in {\mathcal {I}}$ and $n \in 0,\ldots ,k$ we create new variables *i*[*n*] with the same range as *i*. For each variable $v \in {\mathcal {O}}$ and $n \in 0,\ldots ,k$ as well as each $v \in {\mathcal {X}}$ and $n \in 0,\ldots ,k+1$, we create new variables $v^{\top }[n]$ and $v^{\bot }[n]$ with the same ranges as *v*. For a formula $\psi$, let $\psi ^{\top }_n$ be the formula that results from replacing each variable $v \in {\mathcal {I}} \cup {\mathcal {O}} \cup {\mathcal {X}} \setminus \{\mathsf {mut} \}$ with $v^{\top }[n]$, each variable $v' \in {\mathcal {X}} '$ with $v^{\top }[n+1]$, and $\mathsf {mut}, \mathsf {mut} '$ with $\top$. Likewise, $\psi ^{\bot }_n$ is defined.

We can encode trace prefixes up to *k* steps of the original respectively mutated STS as models of the following formulas with free input, output, and state variables (original respectively mutated versions):

$$\begin{aligned} \phi _{otr}^{{\mathcal {S}} ^{c(m)}} {\mathop {=}\limits ^{\tiny def }}\alpha ^{\bot }_0 \wedge \bigwedge _{0 \le n \le k} \delta ^{\bot }_n \quad \quad \phi _{mtr}^{{\mathcal {S}} ^{c(m)}} {\mathop {=}\limits ^{\tiny def }}\alpha ^{\top }_0 \wedge \bigwedge _{0 \le n \le k} \delta ^{\top }_n \end{aligned}$$

We encode potential killing linear tests of length *k* as models of the following formula with free inputs, original output, and state variables:

$$\begin{aligned} \begin{aligned}&\phi _{pk}^{{\mathcal {S}} ^{c(m)}} {\mathop {=}\limits ^{\tiny def }}\forall o^{\top }_0[0],\ldots ,o^{\top }_{m_o}[k], x^{\top }_0[0],\ldots ,x^{\top }_{m_x}[k+1].\\&\quad \alpha ^{\bot }_0 \wedge \bigwedge _{0 \le n \le k} \delta ^{\bot }_n \wedge \big ((\alpha ^{\top }_0 \wedge \bigwedge _{0 \le n \le k} \delta ^{\top }_n) \\&\quad \rightarrow \bigvee _{0 \le j \le m_o} o^{\top }_j[k] \ne o^{\bot }_j[k]\big ) \end{aligned} \end{aligned}$$

Likewise, we encode definitely killing linear tests of length *k* as models of the following formula with free inputs:

$$\begin{aligned} \begin{aligned}&\phi _{dk}^{{\mathcal {S}} ^{c(m)}} {\mathop {=}\limits ^{\tiny def }}\forall o^{\bot }_0[0],\ldots ,o^{\bot }_{m_o}[k], x^{\top }_0[0],\ldots ,x^{\top }_{m_x}[k+1], \\&\quad o^{\top }_0[0],\ldots ,o^{\top }_{m_o}[k], x^{\top }_0[0],\ldots ,x^{\top }_{m_x}[k+1].\\&\quad (\alpha ^{\top }_0 \wedge \bigwedge _{0 \le n \le k} \delta ^{\top }_n \wedge \alpha ^{\bot }_0 \wedge \bigwedge _{0 \le n \le k} \delta ^{\bot }_n) \\&\quad \rightarrow \bigvee _{0 \le j \le m_o} o^{\top }_j[k] \ne o^{\bot }_j[k] \end{aligned} \end{aligned}$$

In the following proposition, we prove the correctness of the encoding.

#### Proposition 10

Let ${\mathcal {S}} ^{c(m)}$ be a conditional mutant, then

1. $\phi _{otr}^{{\mathcal {S}} ^{c(m)}}$ is satisfiable *iff* there is trace $p \in {\mathcal {T}} ({\mathcal {S}})$ of length at least *k*
2. $\phi _{mtr}^{{\mathcal {S}} ^{c(m)}}$ is satisfiable *iff* there is trace $p \in {\mathcal {T}} ({\mathcal {S}} ^m)$ of length at least *k*
3. $\phi _{pk}^{{\mathcal {S}} ^{c(m)}}$ is sat. *iff* there is a linear test *t* for ${\mathcal {S}}$ of length *k* potentially killing ${\mathcal {S}} ^m$
4. $\phi _{dk}^{{\mathcal {S}} ^{c(m)}}$ is sat. *iff* there is a linear test *t* for ${\mathcal {S}}$ of length *k* definitely killing ${\mathcal {S}} ^m$

#### Proof

Statement 1: A model for $\phi _{otr}({\mathcal {S}} ^{c(m)})$ is an assignment of stepwise copies of input, original output and original state variables up to step *k* that satisfies the initial state predicate and the transition predicate in each step. Clearly, such an assignment corresponds to the prefix of trace $p \in {\mathcal {T}} ({\mathcal {S}})$ of length *k*.

Statement 2 can be shown analogously to Statement 1.

Statement 3: As shown Statement 1, every model of $\phi _{pk}({\mathcal {S}} ^{c(m)})$ encodes a prefix *t* of some trace $p \in {\mathcal {T}} ({\mathcal {S}})$ of length *k* and every such prefix is encoded in a model. Furthermore, every extension of such a model via assignments of the universally quantified mutant output and mutant state variables that encodes a prefix of some trace of $q \in {\mathcal {T}} ({\mathcal {S}} ^m)$ with the same input values as *p* in the first *k* steps satisfies the antecedent of the implication and thus needs to satisfy the consequent. Due to Statement 2 every trace of ${\mathcal {S}} ^m$ is captured via such an assignment. Therefore, there exists a model corresponding to trace prefix *t* of the formula if and only if every trace $q \in {\mathcal {T}} ({\mathcal {S}} ^m)$ with $q\vert _{{{\mathcal {I}}}}[0,k] = t\vert _{{{\mathcal {I}}}}$ is such that $q\vert _{{{\mathcal {O}}}}[k] \ne t\vert _{{{\mathcal {O}}}}[k]$, which is equivalent to *t* potentially killing ${\mathcal {S}} ^m$.

Statement 4 can be shown analogously to Statement 3 with the exception that a model only encodes a sequence of inputs. Extensions of that model of universally quantified variables that satisfy the antecedent encode a trace in the original and a trace in the mutated STS with equal input. Since also the consequent needs to be satisfied by such extensions, their outputs must differ in step *k*, showing that every trace of the original STS with the model’s sequence of inputs is a definitely killing linear test of length *k*. $\square$

#### Example 6

Consider the STS of the beverage machine presented in Example 1.

$$\begin{aligned} \begin{aligned} \alpha {\mathop {=}\limits ^{\tiny def }}&\texttt {out=}\varepsilon \wedge \texttt {wtr=2} \\ \delta {\mathop {=}\limits ^{\tiny def }}&\texttt {in=req} \wedge \texttt {wtr>0} \wedge \texttt {out=coff} \wedge \texttt {wtr'=wtr-1} \vee \\&\texttt {in=req} \wedge \texttt {wtr>0} \wedge \texttt {out=tea} \wedge \texttt {wtr'=wtr-1} \vee \\&\texttt {in=fill} \wedge \texttt {wtr=0} \wedge \lnot \texttt {mut} \wedge \texttt {out=}\varepsilon \wedge \texttt {wtr'=2} \vee \\&\texttt {in=fill} \wedge \texttt {wtr=0} \wedge \texttt {mut} \wedge \texttt {out=}\varepsilon \wedge \texttt {wtr'=1} \vee \\&\texttt {in=}\varepsilon \wedge \texttt {out=}\varepsilon \wedge \texttt {wtr'=wtr} \end{aligned} \end{aligned}$$

We present the initial state predicate and transition predicate for the $j'th$ step for the mutated system. In particular, note that due to conjuncts $\lnot \top$ and $\top$ in place of $\lnot \texttt {mut}$ and $\texttt {mut}$ only the transition corresponding to the mutant is activated.

$$\begin{aligned} \begin{aligned} \alpha ^{\top }_0 {\mathop {=}\limits ^{\tiny def }}&out^{\top }[0]=\varepsilon \wedge wtr^{\top }[0]=2 \\ \delta ^{\top }_j {\mathop {=}\limits ^{\tiny def }}&(in[j]=\texttt {req} \wedge wtr^{\top }[j]> 0 \wedge out^{\top }[j]\\&=\texttt {coff} \wedge out^{\top }[j+1] = out^{\top }[j] - 1) \vee \\&(in[j]=\texttt {req} \wedge wtr^{\top }[j] > 0 \wedge out^{\top }[j]\\&=\texttt {tea} \wedge out^{\top }[j+1] = out^{\top }[j] - 1) \vee \\&(in[j]=\texttt {fill} \wedge wtr^{\top }[j] = 0 \wedge \lnot \top \wedge out^{\top }[j]\\&=\varepsilon \wedge wtr^{\top }[j+1] = 2) \vee \\&(in[j]=\texttt {fill} \wedge wtr^{\top }[j] = 0 \wedge \top \wedge out^{\top }[j]\\&=\varepsilon \wedge wtr^{\top }[j+1] = 1) \vee \\&(in[j]=\varepsilon \wedge out^{\top }[j]\\&=\varepsilon \wedge wtr^{\top }[j+1] = wtr^{\top }[j])\\ \end{aligned} \end{aligned}$$

**Fig. 3:**
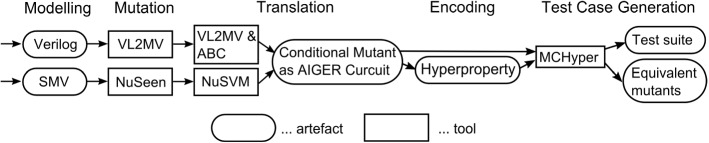
Tool pipeline of our experiments

In order to evaluate the scalability of this method, we encoded a parametrized version of the beverage machine, together with $\phi _{dk}^{{\mathcal {S}} ^{c(m)}}$ in the SMTlib format and gave it to the Z3 SMT solver (version 4.8.7). The benchmark encoding is parametrized with the bound *k* (5 in the running example instance, corresponding to input sequence $\langle$request, request, fill, request, request$\rangle$), which can be controlled via the capacity of the water tank (2 in the running example instance). The encoding and a script to create parametrized versions of it can be found in [47]. We ran this proof of concept demonstration on a virtual machine with one Intel i7 core at 2.8 GHz and 10GB of RAM.

The instance with bound 12 is solved within 20 seconds. After that, there seems to be a steep increase in complexity. For the instance with bound 13, Z3 returns unknown after 7 minutes with an error indicating model-based quantifier instantiation did not find a model after 1000 attempts. Unsurprisingly, the large amount of universal quantification poses a challenging problem to Z3.

## Experiments

In this section, we present an experimental evaluation of the test generation via HyperLTL model checking method. We start by discussing the deployed tool chain. Thereafter, we show a validation of our method on one case study with another model-based mutation testing tool. Finally, we present quantitative results on a broad range of generic models.

### Toolchain

Figure 3 shows the toolchain that we use to produce test suites for models encoded in the modeling languages Verilog and SMV. Verilog models are deterministic while SMV models can be non-deterministic.

*Variable annotation* As a first step, we annotate variables as inputs and outputs. These annotations were added manually for Verilog, and heuristically for SMV (partitioning variables into outputs and inputs).

*Mutation and transformation* We produce conditional mutants via a mutation engine. For Verilog, we implemented our own mutation engine into the open-source Verilog compiler VL2MV [17]. We use standard mutation operators, such as replacing arithmetic operators, Boolean relations. The list of mutation operators used for Verilog can be found in the Table 1. For SMV models, we use the NuSeen SMV framework [7, 8], which includes a mutation engine for SMV models. The mutation operators used for SMV are summarized in Table 2 and explained in detail in [7]. We implemented the non-determinism controlling transformation presented in Sect. 5 into NuSeen and applied it to conditional mutants.

**Table 1:** List of supported Verilog mutation operators ($^*$ marks bit-wise operations)

Type	Mutation
Arithmetic	Exchange binary $+$ and −
	Exchange unary $+$ and −
Relations	Exchange $==$ and $!=$
	Exchange <, $\le$, >, $\ge$
Boolean	Exchange ! and $\sim ^*$
	Drop ! and $\sim ^*$
	Exchange&&,||,&$^*$,$|^*$, $\mathop {xor}$ and $\mathop {xnor}$
Assignments	Exchange $=$ and $<=$
	(Blocking & Non-Blocking Assignment)
Constants	Replace Integer Constant *c* by $0,1,c+1$, and $c-1$
	Replace Bit-Vector Constant by $\vec {0}$, and $\vec {1}$

**Table 2:** List of supported SMV mutation operators

Type	Mutation
Structural	Remove branch in case expression
	Swap branches in case expression
	Remove variable assignment
	Remove variable initialization
	Remove transition constraint
Expressions	Expression negation (*e* is replaced by $\lnot e$)
	Logical operator replacement $( \& ,|,\rightarrow ,\leftrightarrow , \mathop {xor}, \mathop {xnor})$
	Mathematical operator replacement $(+,-,*,/,\mathop {mod})$
	Relational operator replacement $(=,\ne ,<,\le ,>,\ge )$
	Stuck at 0/1 (replace by $\mathsf {false}/ \mathsf {true}$)
	Associative shift $((a | b) \& c$ is replaced with $a | (b \& c))$
Values	Enumeration replacement
	Number replacement
	Digit replacement

**Fig. 4:**
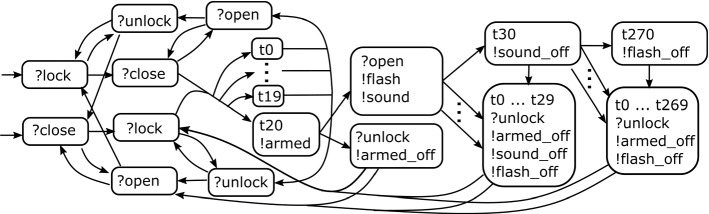
Non-deterministic timed car alarm system model

*Translation* The resulting conditional mutants from both modeling formalisms are translated into AIGER circuits [11]. AIGER circuits are essentially a compact representation for finite models. The formalism is widely used by model checkers. For the translation of Verilog models, VL2MV and the ABC model checker are used. For the translation of SMV models, NuSMV is used.

*Test suite creation* We obtain a test suite via HyperLTL model checking $\lnot \phi _1({\mathcal {I}},{\mathcal {O}})$ on conditional mutants using the MCHyper model checker. Tests are obtained as counter-examples, which are finite prefixes of $\pi$-witnesses to $\phi _1({\mathcal {I}},{\mathcal {O}})$. In case we can not find a counter-example, and use a complete model checking method, the mutant is provably equivalent.

### Car alarm system (CAS) case study

Figure 4 depicts a model of a car alarm system, represented as a labeled transition system, which was studied in the model-based test case generation literature before [2, 4, 27]. Inputs and outputs are marked with leading ’?’ and ’!’ symbols, respectively. The model includes timing sensitive transitions. Discrete time is modeled by hidden propositions $t0,t1,\ldots$ and non-deterministic transitions to states representing that the respective amount of clock ticks have passed. States for a range of time are depicted in compacted form.

The modeled car can be opened, closed, locked, and unlocked. Initially the car is open and unlocked. Once the car is closed and locked, after some time (20 clock ticks in the depicted instantiation) the car enters an armed state. In that armed state, if it is opened before it is unlocked, a visual (flash) and an acoustic (sound) alarm are triggered. After some specified time (30 clock ticks in the depicted instantiation), the visual alarm stops. Then after some more time (270 clock ticks in the depicted instantiation) the acoustic alarm also stops. At any time, the alarms can be turned off by unlocking the car.

We can tune the degree of non-determinism of the model by adjusting the timers of time triggered events. In the following, we discuss mutants for deterministic and non-deterministic cases. Note that we display state-machine representations of the model and its mutants, which are the result of applying mutations to the underlying syntactic description of the model.

#### Deterministic case

In case all timers of time triggered events are 0, the model is deterministic. In this case, we can study mutations on non time-triggered transitions. For example, we can introduce a mutation that disables some transition. In the transition system representation of the model, this amounts to replacing the transition with a self-loop. In a syntactic representation of the model, this amounts to replacing the condition of a branch by $\mathsf {false}$.

**Fig. 5:**
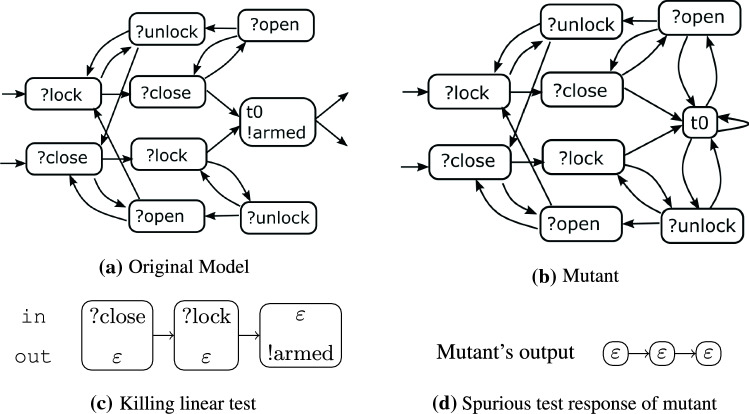
Deterministic mutant of a deterministic model

We depict the relevant parts of a deterministic version of the car alarm system model in Fig. 5a and a mutant with a faulty arming mechanism Figure 5b. The mutant does not enter the armed state after the car is locked and closed. The test depicted in Fig. 5c definitely kills this mutant, as can be seen in the response of the mutant to the test in Fig. 5d.

**Fig. 6:**
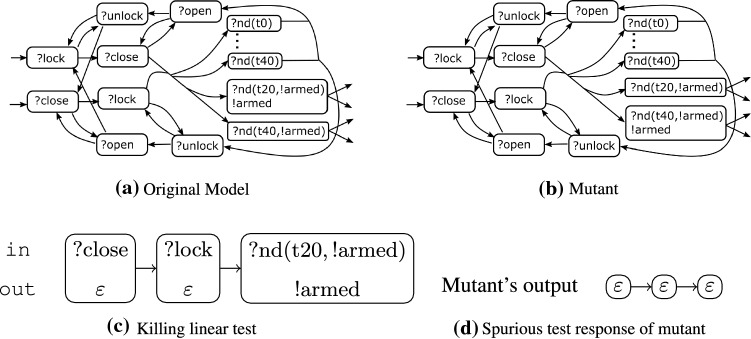
Mutant and model with controllable non-determinism

#### Non-deterministic case

In case some timer of time triggered events is nonzero, the model is non-deterministic. As discussed in Sect. 5, in order to deal with non-deterministic models in practice, we need to make non-determinism controllable. We depict the relevant parts the transformed non-deterministic version of the car alarm system model in Fig. 6a and a mutant that doubles the time trigger for entering the armed state in Fig. 6b. Note that due to transformation making non-deterministim controllable, in contrast to Fig. 4, the time propositions are input propositions. Further note that due to equal input-enabledness, both model and mutant have transitions to states for the whole range of time from 0 to 40 clock ticks, as well as states corresponding to anticipating the armed state after 20 and 40 clock ticks, which differ in observable output between the model and mutant. The test depicted in Fig. 6c kills that mutant, as can be seen in the response of the mutant to the test in Fig. 6d, which controls timing. However, note that the original mutant with uncontrollable non-determinism is only potentially killable, since after inputs ?open and ?close, there is a non-deterministic transitions in the mutant that produces the prescribed output !armed. That is, the test only kills the mutant reliably when timing can be controlled. Although in practice it might be difficult to execute a test that requires to wait an exact amount of time, it should be noticeable whether the time to enter the armed state is twice as much as the expected time.

The car alarm system model can easily be modified to illustrate the mixed determinism case. To this end, a model with all timers being 0 can be compared to one with some timer greater than 0.

**Table 3:** Characteristics of models

Parameters	Verilog				SMV				CAS
	$\mu$	$\sigma$	Min	Max	$\mu$	$\sigma$	Min	Max	
Models	16				76				1
Input	186	310	4	949	9	13	0	88	58
Output	177	299	7	912	4	4	1	28	7
State	16	16	2	40	–	–	–	–	–
Gates	4207	8309	98	25193	189	210	7	1015	1409
$\varDelta$ Gates (%)	3	3	0.1	10	8	8	0.3	35	0.9
Mutants	260	236	43	774	535	1042	1	6304	3057

#### Test suite evaluation

We evaluated the strength and correctness of the test suite created using the methods, toolchain, and SMV mutation operators presented in this work via the model-based mutation testing tool MoMuT [1, 27] on a non-deterministic version of the car alarm system. To this end, we manually formulated the model both in SMV and as an action system, which is the native modeling language for MoMuT. The two modeling mechanisms induce two separate sets of mutants, i.e.,, those induced by SMV mutation operators described above and those induced by action system mutation operators, which are out of scope of this work, but are described in [27]. MoMuT can evaluate a test suite (created via our toolchain and using SMV mutations) by computing its mutation score—the ratio of killed to the total number of mutants—with respect to action system mutations on a given action system model.

This procedure evaluates our test suite in two ways. Firstly, it shows that the tests are well formed, since MoMuT does not reject them. Secondly, it shows that the test suite is able to kill mutants other than those it was created from, which is important, because it suggests that the test suite is also able to detect faults in implementations.

We created a test suite consisting of 61 tests, using the toolchain presented in Sect. 6.1 and the mutation operators presented in Table 2. We automatically map the resulting test suite to the test format accepted by MoMuT and automatically remove redundant tests. A test is redundant if its string representation is a prefix of another test. For the test suite, MoMuT measures a mutation score of 91% on 439 action system mutants. In comparison, the test suite achieves a mutation score of 61% on 3057 SMV mutants for which is was created. These results highlight that the mutation score is relative to the mutation operators used. On this model, the SMV mutation operators produce a lot more equivalent mutants than the action system mutation operators. Further characteristics of the resulting test suite are presented in the following Sect. 6.1.

Finally, we created a separate test suite using MoMuT with its default settings on the action system model. The resulting test suite consisted of 6 tests that kill 90% of the action system mutants, which were 8 mutants less than the test suite created via hyperproperties. The test suite created by MoMuT is more compact, because it was created directly for action system mutants instead of the larger number of SMV mutants. This result shows that hyperproperty model checking-based test generation is well suited to kill a large array of mutants, while mature mutation testing tools are able to create more compact test suites that kill a large proportion of the mutants. A combined use of both techniques is one interesting future direction of this work.

### Quantitative experiments

We present experiments on a series of benchmark that demonstrate the versatility and scalability properties of generating test suites via hyperproperty model checking. The experiments were run in parallel on a machine with an Intel(R) Xeon(R) CPU at 2.00GHz, 60 cores, and 252GB RAM. We used 16 Verilog models which are presented in [31], as well as models from opencores.org. Furthermore, we used 76 SMV models that were also used in [7]. Finally, we used the SMV formalism of CAS. All models are available in [47]. Verilog and SMV experiments were run using property driven reachability based model checking with a time limit of 1 hour. Property-driven reachability based model checking did not perform well for CAS, for which we therefore switched to bounded model checking with a depth limit of 100. All reported values are rounded to the first significant digit.

**Table 4:** Experimental results

Metrics	Verilog				SMV				CAS
	$\mu$	$\sigma$	Min	Max	$\mu$	$\sigma$	Min	Max	
Killed (%)	57	33	5	99	65	31	0	100	62
Avg. test length	4	2	2	8	15	58	4	462	6
Max test length	22	50	3	207	187	1279	4	10006	9
Avg. runtime (s)	83	268	0.01	1068	1	5	–	47	8
Equivalent (%)	33	32	0	95	35	31	0	100	0
Avg. runtime (s)	45	120	–	352	1	2	–	15	–
Timeout (%)	10	27	0	86	0	0	0	0	38
Total time (h/s)	69	169	3s	620	0.4	1	0.1s	7	1

*Characteristics of models* Table 3 present characteristics of the models. For Verilog and SMV, we present average ($\mu$), standard deviation $(\sigma )$, minimum (Min), and maximum (Max) measures per model over the set of models. For CAS we report the values for that single model. We report the size of the circuits in terms of the number of **Input**-, **Output**-, **State** variables as well as the number of *And* **Gates**, which corresponds to the size of the transition relation of the model. Furthermore, in row $\varDelta$ **Gates (%)**, we report the average absolute size difference (in % of number of Gates) of the conditional mutant and the original model, where the average is over all mutants. Finally, **Mutants** shows the number of the mutants that are generated and analyzed for the models.

We can observe that our method is able to handle models of respectable size, reaching thousands of gates. Furthermore, $\varDelta$ Gates of the conditional mutants is relatively low. Thus, conditional mutants allow us to compactly encode the original and mutated model in one model. Hyperproperties enable us to refer to and juxtapose traces from the original and mutated model, respectively. Classical temporal logic does not enable the comparison of different traces. Therefore, mutation analysis by model checking classical temporal logic necessitates strictly separating traces of the original and the mutated model, resulting in a quadratic blowup in the size of the input to the classical model-checker, compared to the size of the input to the hyperproperty model-checker.

*Model checking results* Table 4 summarizes the quantitative results of our experiments. Similarly to above, for Verilog and SMV, we present average ($\mu$), standard deviation $(\sigma )$, minimum (Min), and maximum (Max) measures per model over the set of models as well as the respective values for the single CAS model. The quantitative metrics we use for evaluating our test generation approach are **Killed (%)** the percentage of killed mutants, **Equivalent (%)** the percentage of equivalent mutants, **Avg. test length**, **Max test length**, the average respectively maximal test case length for tests produced for each killed mutant, as well as **Avg. Runtime (s)** the amount of model checking time per killed respectively equivalent mutant. Furthermore, we report **Timeout (%)** the percentage of mutants exceeding the time limit or BMC depth bound. For Verilog and SMV the time limit was 1 hour. For CAS the depth limit was 100 transitions.

Finally, we report **Total time (h/s)** the total time for test suite creation per model, including timeouts, in hours or seconds for very small models. The total time is the sum of the per mutant model checking times, i.e., assumes sequential test suite creation. However, since mutants are model checked independently, the process can easily be parallelized, which drastically reduces the total time needed to create a test suite for a model, typically from hours to a few minutes. The times of the Verilog benchmark suite are dominated by two instances of the secure hashing algorithm (SHA), which are inherently hard cases for model checking.

We can see that the test suite creation times are in the realm of a few hours, which collapses to minutes when model checking instances in parallel. However, the timing measures really say more about the underlying model checking methods than our proposed technique of mutation testing via hyperporperties. Furthermore, we want to stress that our method is agnostic to which variant of model checking (e.g.,, property driven reachability, or bounded model checking) is used. As discussed above, for CAS switching from one method to the other made a big difference.

The mutation scores average is around 60% for all models. It is interesting to notice that the scores of the Verilog and SMV models are similar on average, although we use a different mutation scheme for the types of models. Again, the mutation score says more about the mutation scheme than our proposed technique. Notice that we can only claim to report the mutation score, because, besides CAS, we used a complete model checking method (property-driven reachability). That is, in case, for example, 60% of the mutants were killed and no timeouts occurred, then 40% of the mutants are provably equivalent. In contrast, incomplete methods for mutation analysis can only ever report lower bounds of the mutation score.

The Verilog model with the highest percentage of killed mutants (99%) is a deterministic Verilog version of an car alarm system without time. In total, that model has 104 mutants of which 103 were killed and one was equivalent. The Verilog model with the least percentage of killed mutants (0%) is an encoding of the SHA encryption algorithm. Such models are notoriously difficult cases for model-checking methods. Out of its 687 total mutants, 592 mutants (86%) timed out during model checking and the remaining 95 mutants (14%) were killed.

Multiple SMV models correspond to the highest percentage of killed mutants (100%), including a model of digital adder with 36 mutants. All models with 100% killing percentage are rather small. However, one model of a Java array implementation with 2554 mutants has a killing percentage of 97%. Likewise, there are multiple SMV models with the highest number of equivalent mutants (100%), including, for example, a model of the rock–paper–scissors game with 187 mutants.

Finally, as discussed above, the 61.7% of CAS translate to 91% mutation score on a different set of mutants. This indicates that the failure detection capability of the produced test suites is well, which ultimately can only be measured by deploying the test cases on real systems.

## Related work

### Hyperproperties

Hyperproperties were originally introduced to formally express security properties, such as non-interference, in [19]. The paper works out the theoretical foundations of hyperproperties and contrasts them to classical trace properties. Traditionally, hyperproperties were formulated and used in a case by case fashion, see for example [41, 45, 53].

In order to generalize these approaches and to enable rigorous study of hyperproperties, logics for hyperproperties were developed [18, 20]. In particular, HyperLTL and HyperCTL*, which are hyperproperty sensitive extensions of classic temporal logics, are used in this work. The expressive power of HyperLTL was characterized to be equivalent to first order logic over disjoint copies of the natural numbers and a restricted type of quantification [32].

Furthermore, the satisfiability- [28, 29], monitoring- [15, 30], and model-checking [31] problems for HyperLTL were tackled. The initial lack of model checking techniques for HyperLTL formulas with quantifier alternation was recently addressed in [21] via a combination of reactive synthesis and model checking of quantifier alternation free HyperLTL formulas. Unfortunately, the respective version of the HyperLTL model checker MCHyper is currently only available in a Web-based version.

### Model checking-based test generation

A number of test case generation techniques are based on model checking; a survey is provided in [35]. The approach has been demonstrated to scale to the industrial setting in [24]. In [33] a thorough evaluation and comparison of different model checkers applied to the test generation problem over multiple modeling formalisms is presented.

Most model checking based test generation target, in comparison with our work, different coverage metrics and/or abstraction levels, such as structural coverage criteria for Java programs [54] and RSML models [51] or information/data flow criteria for extended finite state machines [38].

However, mutation testing via model checking has been explored as well. For example, [36] presents an approach to formulate mutation killing via trap properties. Trap properties are conditions that, if satisfied, indicate a killed mutant. In contrast, our approach directly targets the input / output behavior of the model and does not require to formulate model specific trap properties.

Mutation based test case generation via module checking is proposed in [13]. The theoretical framework of this work is similar to ours, but builds on module checking instead of hyperproperties. Moreover, no implementation or experimental evaluation is provided, leaving the practical applicability of the approach open.

In [5] an approach for mutation based test case generation of timed automata is presented. The test case generation problem is reduced to a language inclusion problem, which is solved via bounded SMT model checking. Similarly, [25] presents an approach to mutation-based test generation via model checking for embedded software. The authors combine the original model, mutants, and mutation detection monitors into one timed automaton model. A reachability property is then model checked over this combined model to generate killing test cases. In contrast to our work, in presence of non-determinism, the proposed encodings of mutation killing can not differentiate between potential and definite killing.

In [34] model checking based test generation is used to check requirements property focused coverage criteria, such as mutation of properties. Similarly, [12] presents an approach to create mutated requirements and use model checking of SMV models to identify models that fulfill the faulty requirements. Our work is orthogonal to such approaches, since we consider test generation over mutations of the system instead of the property.

### Symbolic test generation

The authors of [4] present an approach to mutation based test generation for action system models via symbolic refinement condition. The refinement condition as well as sets of reachable states are iteratively computed by solving SMT problems. While this work offers an interesting practical solution for action systems, our approach targets a larger class of systems that can be encoded as symbolic transition systems. In a similar fashion, the MuAlloy [55] framework enables model-based mutation testing for Alloy models using SAT solving. In this work, the model, as well as killing conditions, are encoded into a SAT formula and solved using the Alloy framework.

In contrast to the latter two approaches, we encode only the killing conditions into a formula and leave encoding of the transition system to the model checker. Therefore, our approach is more flexible and more likely to be applicable in other domains. We demonstrate this by producing test cases for models encoded in two different modeling languages.

Symbolic methods for weak mutation coverage are proposed in [10] and [9]. The former work describes the use of dynamic symbolic execution for weakly killing mutants. The latter work describes a sound and incomplete method for detecting equivalent weak mutants. The considered coverage criterion in both works is weak mutation, which, unlike the strong mutation coverage criterion considered in this work, can be encoded as a safety traceproperty. However, both methods could be used in conjunction with our method. Dynamic symbolic execution could be used to first weakly kill mutants and thereafter strongly kill them via hyperproperty model checking. Equivalent weak mutants can be detected with the methods of [9] to prune the candidate space of potentially strongly killable mutants for hyperpropery model checking.

### Semantics of mutation coverage

A comprehensive survey of mutation testing in multiple settings is presented in [40]. The foundational ideas of mutation testing were first presented in [23] and [37]. What we consider the original definition of mutation coverage was presented in [16], which considers mutants of deterministic programs. In this original definition, a mutant is killed if it produces different output to the original. However, in different settings, such as in model-based testing or in the presence of non-determinism, this simple definition is not satisfactory, since in the former case tests are created from an abstraction of the system under test and in the latter case different non-deterministic behavior has to be differentiated from real faults. While the basic notion of introducing faults and seeking differences in outcome remains the same, different system abstraction levels or test requirements might result in different semantic definitions of mutation coverage. One goal of our work is to provide rigorous semantics for non-deterministic systems that are applicable to a large class of settings.

Refinement relations between systems are used to define mutation coverage in [4, 27] for action systems or in [3] for the Unifying Theory of Programming. The resulting killing criteria are essentially equivalent to potential killing, since for non-deterministic systems, a single spurious output constitutes a killed mutant.

In [14], mutation coverage is defined for communicating extended finite state machines by comparing all possible input/output sequences following some input sequence of original and mutant. The considered notion of killing is similar, yet different to the potential or definite killing considered in this work, since it considers all inputs and outputs after some prefix inputs, whereas we consider all outputs after a sequence of inputs.

A unified framework for defining multiple test coverage criteria, including weak mutation and hyperproperties such as unique-cause MCDC, is proposed in [44] . While strong mutation is not expressible in this framework, applying hyperproperty model checking to the proposed framework is interesting future work.

## Conclusion

Our formalization of mutation killability in terms of hyperproperties provides rigorous semantics, in particular in presence of non-determinism, and enables the automated model-based generation of tests using an off-the-shelf model checker. We overcome limitations of currently available hyperproperty model checking tooling infrastructure by providing methods to create test cases for non-deterministic models via a transformation making non-determinism controllable and an SMT encoding of killability properties that require quantifier alternation. Furthermore, we evaluated our approach on publicly available SMV and Verilog models, demonstrating that the approach is versatile and scalable.

## Footnotes

1 Finite domains can be characterized using binary encodings; infinite domains require an extension of our formalism in Sect. 2.2 with equality that is omitted for the sake of simplicity.
